# Evaluation of morpho-physiological responses to drought and salt stress in two ornamental alternatives to Invasive *Ligustrum sinense* Lour.

**DOI:** 10.3389/fpls.2026.1770722

**Published:** 2026-04-24

**Authors:** Giulia Daniele, Matteo Caser, Federica Larcher

**Affiliations:** 1 Department of Agricultural, Forest and Food Sciences, University of Torino, Torino, Italy; 2 NBFC, National Biodiversity Future Center, Palermo, Italy

**Keywords:** urban horticulture, salinity stress, water stress, invasive species, *Ligustrum*

## Abstract

Urban environments are increasingly subject to anthropogenic pressures and climate change-induced stresses such as salinity and drought. Urban greening initiatives require the selection of ornamental species that are increasingly adapted to the unique challenges of cities. This study evaluates the response to salt and drought stress of two potential alternatives to the non-native invasive species *Ligustrum sinense* Lour.: *Ligustrum vulgare* L. (European native), and *Ligustrum japonicum* Thunb. ‘Texanum’ (non-invasive ornamental cultivar). A total of 270 plants, approximately 25 cm in height, including 90 plants each of *L. sinense*. *L. japonicum* ‘Texanum’, and *L. vulgare* were placed in a growth chamber and grown under controlled conditions from March 2024 to June 2024. The plants were subjected to one of two NaCl treatments, corresponding to 150 mM (moderate salt stress) and 300 mM (severe salt stress) or deionized water (control). Additionally, two irrigation levels were implemented, specifically 30% (severe drought stress) and 60% of the pot capacity (moderate drought stress), while the control group received 90% of the pot capacity. All three species showed intolerance to severe drought stress and its combined effect with moderate and severe salt stress. *Ligustrum sinense* survived only to moderate drought stress (60%). Moderate drought stress negatively affected 3D leaf area, digital biomass, and chlorophyll content, as well as dry biomass of shoots and roots, and compromised root development. In *L. japonicum* ‘Texanum’, drought and salinity stresses, applied individually or in combination, led to a significant reduction in 3D leaf area and digital biomass. Salinity significantly affected water band index, particularly at high levels and under combined stress. Shoot dry biomass declined under all stresses, while dry root biomass was unaffected. In *L. vulgare*, salinity was the main limiting factor for 3D leaf area and digital biomass, particularly under combined salt and drought conditions, causing reductions in NDVI, chlorophyll content, leaf water status, and pigment balance, indicating accelerated leaf senescence. Salinity and combined stresses most strongly affected shoot dry biomass, and also decreased root dry biomass and architecture. These findings support the use of these species as alternatives in European urban greening efforts.

## Graphical Abstract

**Figure:**
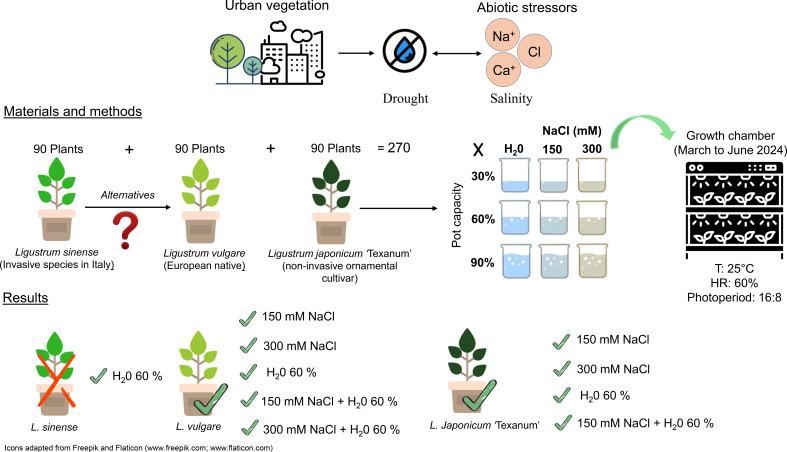

## 1 Introduction

Growing anthropogenic pressure reduces the resilience of urban environments, leading to negative environmental and socioeconomic impacts (European Environment Agency, 2022) and causing a reduction in both the quantity and quality of urban green spaces (Colding et al., 2020; Semeraro et al., 2021). They are characterized by vegetation that can help limit these effects by providing important ecosystem services (ES), such as mitigating the urban heat island effect (Schwaab et al., 2021), reducing stormwater runoff (Dowtin et al., 2023), enhancing air quality (Riondato et al., 2020), promoting biodiversity (Toscano et al., 2025), and improving human well-being (Semeraro et al., 2021). Since the COVID-19 pandemic, public awareness of the role of urban green areas in supporting overall well-being has increased, highlighting the growing need for them in urban settings (Larcher et al., 2021).

However, the survival of ornamental vegetation in urban environments is threatened by numerous biotic and abiotic stressors (Kisvarga et al., 2023). In many European cities, soil salinization and drought are two main abiotic stressors (Cekstere et al., 2020; Franceschi et al., 2023; Łuczak et al., 2021), resulting from the interaction between human activities and climatic factors (Shukla et al., 2019). Among human activities, the use of de-icing salts on roads (Victorovna and Aleksandrovich, 2021) and alternative water sources, such as reclaimed water for landscape irrigation, contribute to increasing salt concentrations in urban soils (Zalacáin et al., 2019). These processes are amplified by climate change due to rising temperatures and increased precipitation, alternating with periods of prolonged drought (Shukla et al., 2019; Chaudhry and Sidhu, 2022).

Nowadays, water scarcity and overuse of available resources are emerging problems affecting about one-third of Europe ^1^, most notably in Mediterranean countries, but also affecting northern regions (Kazmierczak et al., 2020; Rocha et al., 2020). The reuse of treated reclaimed water is a growing practice already permitted in many EU Countries, including Italy, Germany, Greece, and Portugal. This practice contributes significantly to water sustainability ^2^^, ^^3^, but can increase the concentrations of K^+^, Na^+^, Mg^2+^, Ca^2+^and Cl^-^, increasing salinity and limiting plant growth (Vivaldi et al., 2019). Maximum salinity thresholds vary by Country; for example, in Italy, the electrical conductivity (EC) value must not exceed 3 dS/m (Santos et al., 2023).

The interaction between anthropogenic and climatic factors involves not only the individual effects of water and salt stress but also their combined action, which compromises the growth and survival of many ornamental species in urban environments (Baraldi et al., 2019; Shukla et al., 2019; Toscano et al., 2021). Although the individual effects of these stressors have been extensively investigated (Szekely-Varga et al., 2020; Dadach et al., 2021; Toscano et al., 2021), studies on their combined effects are still underrepresented (Rivero et al., 2022). Understanding these interactions is crucial for improving the resilience of urban ecosystems (Kisvarga et al., 2023). Their effects can be quantified using a range of morphological, physiological, and biochemical parameters (Toscano et al., 2021). Numerous studies have confirmed that plants respond negatively to both drought and salt stress, and their combination typically intensifies morpho-physiological damage (Sharif et al., 2018; Angon et al., 2022). A common response to drought stress, also observed under salt stress and their combined occurrence, is a reduction in growth (García-Caparrós and Lao, 2018; Toscano et al., 2021; Balasubramaniam et al., 2023; Mircea et al., 2023a). Drought stress typically leads to a decrease in leaf number and size, often accompanied by increased leaf thickness (Blanco-Sánchez et al., 2023; Mircea et al., 2023a), a significant decrease in photosynthetic rate and stomatal conductance (Dos Santos et al., 2024), and inhibition of root development (Kang et al., 2022). Similarly, salt stress compromises plant performance by reducing chlorophyll content, stomatal conductance, and photosynthetic efficiency, as well as decreasing fresh weight, leaf area, nutrient uptake, and overall visual quality (García-Caparrós and Lao, 2018; Ors et al., 2021). When both stresses occur simultaneously, their negative effects tend to intensify (Sahin et al., 2018), leading to a more severe decline in photosynthetic activity, greater fresh weight loss, and further growth inhibition (Ors et al., 2021; Toscano et al., 2021).

In urban contexts, several effects of human activities not only pose a threat to the growth of ornamental plants but also increase the vulnerability of these habitats to biological invasions (Gallardo et al., 2015; Dullinger et al., 2017). Among the various human activities, the ornamental plant trade is one of the main pathways for the introduction of non-native plant species (Van Kleunen et al., 2018), some of which have become invasive (Blackburn et al., 2011) and are cultivated in many urban areas such as public parks, private gardens, and tree-lined avenues (Čeplová et al., 2017; Khapugin et al., 2023). The use of invasive plants poses a risk to natural habitats (Charpentier et al., 2020), the historical and cultural heritage of cities (Celesti-Grapow and Ricotta, 2021), and human health (Rodríguez et al., 2021). To limit the spread of invasive species and protect ecosystem integrity, many European Countries have established official lists of invasive plants subject to restrictions and control measures in certain regions. Among these, a notable invasive species in Europe, classified as invasive and regulated in some regions of Italy (Piedmont and Lombardy) (Brundu et al., 2020), is *Ligustrum sinense* Lour. (Chinese privet, Fam. Oleaceae). *L. sinense* is a shrub that was introduced from East Asia to Europe in 1852 as an ornamental species (Banfi and Galasso, 2010). It is tolerant to various environmental stress factors, with high phenotypic plasticity of the leaves, a strong capacity for asexual reproduction, and allelopathic effects that contribute to its invasiveness (Pokswinski, 2008). The genus *Ligustrum* spp. is important in the ornamental industry ^4^. Two species, *Ligustrum japonicum* Thunb. ‘Texanum’ (non-native and non-invasive ornamental cultivar) ^5^ and *Ligustrum vulgare* L., a European native^6^, are widely available in the European ornamental market ^7^ and offer valuable benefits that should be considered when designing urban green spaces. Considering the increasing anthropogenic pressures and evident effects of climate change, it is important to consider the use of alternative plant species that are resilient to urban conditions (Daniele et al., 2024). This involves adopting a more ecologically oriented aesthetic that favors native and non-native non-invasive species (Daniele et al., 2023; Tartaglia and Aronson, 2024). Deepening their adaptation to urban conditions is crucial for creating resilient and sustainable cities. In this context, the research conducted as part of the National Biodiversity Future Center (NBFC) project funded by the National Recovery and Resilience Plan (NRRP) aimed to identify alternative ornamental plants to invasive species for urban greening strategies (e.g., urban forest). Accordingly, the morpho-physiological responses to drought stress, salt stress, and their combined effects were analyzed by comparing the invasive species *L. sinense* with *L. vulgare* and *L. japonicum* ‘Texanum’. Based on the high adaptability of invasive species to different unfavorable urban conditions (Montagnani et al., 2026) and proven tolerance to salinity (Caser et al., 2011), *L. sinense* is expected to show significantly higher adaptation strategies. We also hypothesized that the combined effects of drought and salinity stress would have a more pronounced negative impact on morphophysiological responses than either stress considered individually. More severe levels of water and salt stress, applied individually, will produce more negative effects. In particular, it is hypothesized that salt stress will produce more severe results than water stress due to ionic toxicity. The findings of this study will be valuable for validating the suitability of *L. vulgare* and *L. japonicum* ‘Texanum’ as alternatives to the invasive *L. sinense* for urban greening.

## 2 Materials and methods

### 2.1 Plant materials

In March 2024, 270 two-year-old plants, approximately 25 cm in height, were collected from the Purpurea plant nursery by Alberto Peyron (Piobesi Torinese, Piedmont, Italy, 44°56’12 “N Lat. 7°35’42 “E Long.; 233 m s.l.m). The plants were chosen two years old because the experiment was conducted in a growth chamber, where the available space was limited. A total of 90 plants, each of *L. sinense* (0.315 L pots), *L. japonicum* ‘Texanum’ (0.417 L pots), and *L. vulgare* (0.637 L pots), were cultivated in polyethylene pots filled with a substrate characterized by 49.92% dry substance, 30.63% volatile substances, pH 5.30, total nitrogen (N) of 1.99%, total carbon of 20.90%, phosphorus (P) of 3911.75 mg/kg, and total P of 0.39%. In each pot, 7 g of slow-release fertilizer (OSMOCOTE PRO 18:9:10 + 2MgO) was added. Plants were maintained in a growth chamber at the Department of Agricultural, Forestry and Food Sciences of the University of Turin, Italy. The microclimatic conditions inside the growth chamber provided an air temperature of 25 °C, a photoperiod of 16:8 with LED lights red 65% (660 nm), blue 20% (450 nm), and green 15% (between 500 and 600 nm), Ambralight^®^) + NEON, relative humidity (RH) of 60%, and a Photosynthetically Active Radiation (PAR) of 400 μmol m^-2^ s^-1^. After the acclimatization period, the experiment started on March 18, 2024. *Ligustrum* spp. were chosen because of their multiple ecosystem services (**Table 1**), which make them particularly suitable for urban environments. In addition, their botanical and ornamental characteristics, such as flower, fruit, and leaf production, justify their selection for enhancing urban green spaces (photographic documentation of species and cultivars can be viewed on the official website of the Royal Horticultural Society (RHS)^7^.

**Table 1:** List of the species considered in the study, indicating their scientific name, status, origin, plant form, flowering period, and ecosystem services.

Scientific name	Status	Origin	Plants form	Flowering period	Ecosystem services	References
*Ligustrum sinense*	Invasive	Est Asia	Late deciduous shrub	Summer	_	(Royal Horticultural Society^8^; Brundu et al., 2020; Banfi and Galasso, 2010)
*Ligustrum vulgare*	Native	Europe	Deciduous or semi-evergreen	Summer	Mitigation of air quality and its importance for pollinators	(Royal Horticultural Society^9^; Tkachenko et al., 2023; Sandu et al., 2023)
*Ligustrum japonicum* ‘Texanum’	Non-invasive ornamental cultivar	Cina-Asia orientale	Evergreen shrub	June-August	Low allergenicity; year-round coverage; pollution tolerance; Pb phytoremediation	(Royal Horticultural Society^10^; Cariñanos et al., 2017; Kim et al., 2015; Abdollahpour et al., 2022)

### 2.2 Experimental design and treatments

The experimental design was a randomized block design with three factors, nine treatments, and ten replicates per treatment (**Table 2**). The first factor, salt application, involved three concentrations: 0, 150, and 300 mM NaCl (with corresponding electrical conductivity (EC) values of 0, 15.54, and 29.83 dS/m, respectively. The thresholds adopted were based on the results of Caser et al. (2011) on *Ligustrum* sp., extending the salinity range up to 300 mM NaCl. Although these concentrations are high in most common scenarios, strong anthropogenic pressure can cause salinity hotspots, with concentrations reaching 300 mM, as reported in non-European contexts (Shannon et al., 2020). Furthermore, in the literature, these conditions are commonly applied to simulate urban salt stress conditions in ornamental plants (Gugliuzza et al., 2023). The second factor, drought stress, included three levels of pot capacity: control with 90% pot capacity (PC), 60% PC (moderate deficit regime), and 30% PC (severe deficit regime). The water content remained constant throughout the experiment. The gravimetric determination of the water content was performed by saturating the soil samples with water and then allowing drainage to remove excess water. Pot capacity was calculated using the equation of Paquin and Mehuys (1980), based on a gravimetric approach. In the first week, three salt treatments were applied using 1 L solutions delivered through manual irrigation to mimic urban watering practices. In the subsequent weeks, the three levels of pot capacity soil moisture were maintained by hand-irrigating with deionized water, and each container was weighed twice a week until the end of the experiment.

**Table 2:** The experimental treatments included salt stress, pot capacity (%), and their combination, for a total of nine distinct treatments.

Treatments	C	S1	S2	W1	W2	S1W1	S1W2	S2W1	S2W2
Salt stress (mM)	0	150	300	0	0	150	150	300	300
Pot capacity (%)	90	90	90	30	60	30	60	30	60

These included C (control, 90% pot capacity); S1 (150 mM NaCl); S2 (300 mM NaCl); W1 (30% pot capacity); W2 (60% pot capacity); S1W1 (150 mM NaCl + 30% pot capacity); S1W2 (150 mM of NaCl + 60% pot capacity); S2W1 (300 mM of NaCl + 30% pot capacity); S2W2 (300 mM of NaCl + 60% pot capacity).

### 2.3 Morphological measurements

**Figure 1:**
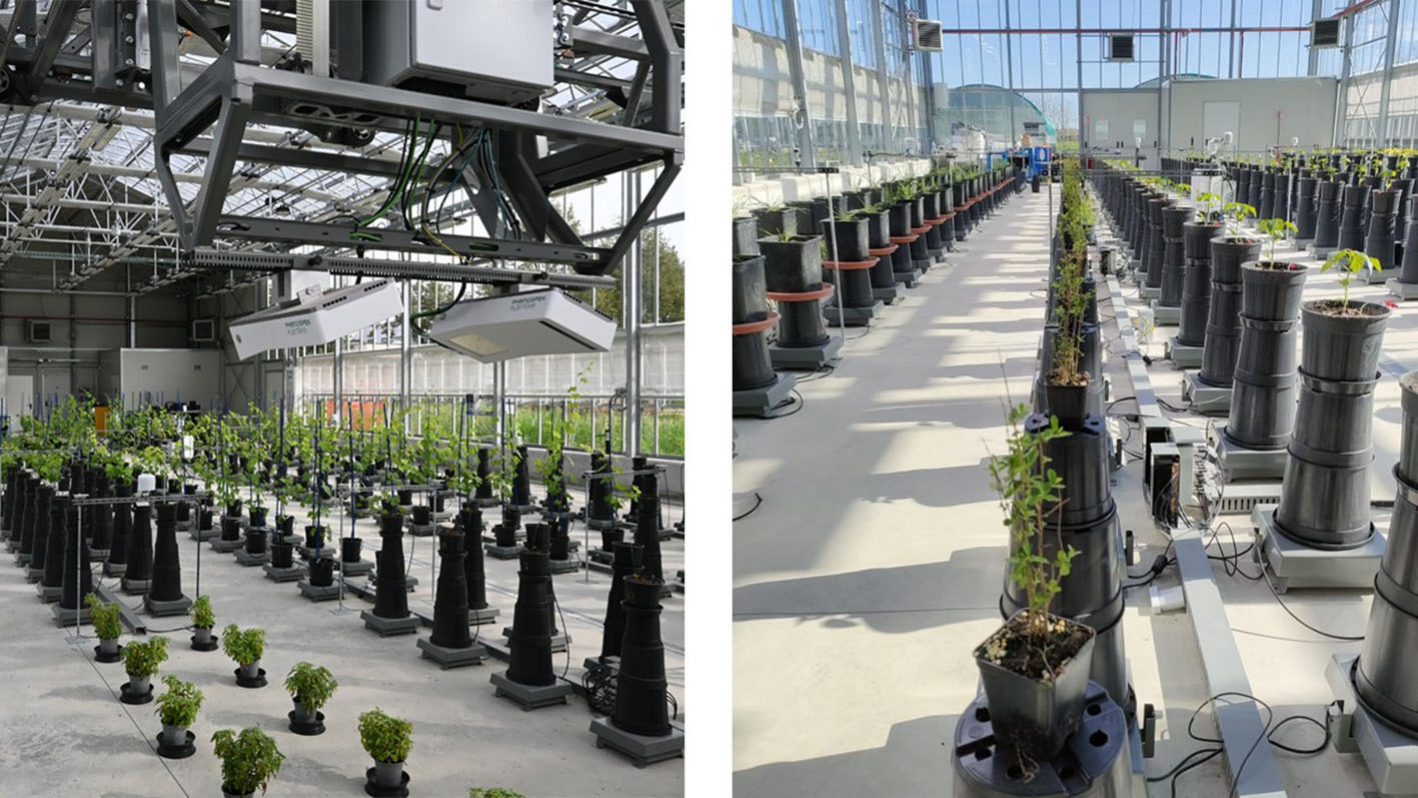
Phenotyping platform PhenoPlant at the Department of Agricultural, Forestry, and Food Sciences, Turin, Italy. In the image on the right, the *Ligustrum vulgare* plants from our research.

Morphological measurements, including three-dimensional leaf area (3D mm²), digital biomass (mm³), and average plant height (mm), were obtained using the advanced phenotyping platform PhenoPlant, at the University of Turin, DiSAFA (Department of Agricultural, Forestry, and Food Sciences, Turin, Italy) (PhenoPlant, the plant phenotyping platform of the Department of Agricultural, Forest and Food Sciences of the University of Turin)^11^. The PhenoPlant platform is an automated greenhouse that uses two PlantEye F500 sensors (Phenospex^®^ BV, Netherlands) with a multispectral camera, lighting unit, and single-line laser to perform 3D reconstruction of the plant, providing morphological parameters and spectral indices (**Figure 1**). Measurements were taken at the midpoint and end of the experiment.

### 2.4 Vegetative index

PhenoPlant measured the green leaf index (GLI), normalized difference vegetation index (NDVI), normalized pigment chlorophyll index (NPCI), and plant senescence reflectance index (PSRI) at the midpoint and the end of the experiment. Using the NaturaSpec portable spectroradiometer RS-5400^®^, the simple ratio (SR), enhanced vegetation index (EVI), soil-adjusted vegetation index (SAVI), modified soil-adjusted vegetation index (MSAVI2), photochemical reflectance index (PRI), water band index (WBI, 900/970 nm), and normalized difference water index (NDWI) were considered. For the vegetation index measurements taken with the spectroradiometer, only the tables with values at T60 (Time 60) and T90 (Time 90) are shown to align the time of measurements taken with the PhenoPlant platform.

### 2.5 Shoot and root dry biomass, and root index

On day 91, all remaining shoot material was severed at the root collar, weighed, placed in paper bags, and dried in a thermoventilated oven at 65°C for one week. Roots were dried separately in a growth chamber at 25°C for one week. Dry biomass values were expressed in grams (g). To assess the effects of the treatments on root development after 90 days, all plants were removed from the pots, and root growth was evaluated using a scale from 0 to 3: 0 = 0–25%, 1 = 26–50%, 2 = 51–75%, and 3 = 76–100% of the pot volume colonized by the roots. Roots and shoots respond differently to stress, so they were separated to allow for a more accurate assessment of their respective responses. The results show only the values for the treatments that survived.

### 2.6 Physiological measurements

A SPAD-502 chlorophyll meter was used to measure the relative chlorophyll content of the leaves of plants in all treatments. stomatal conductance (*g_s_*, mol m^-2^ s^-1^), transpiration rate (E, mmol m^-^²s^-^¹), leaf vapor pressure deficit (VPD_leaf, kPa),Photosystem II efficiency (PhiPS2), and electron transport rate (ETR, µmol e^-^m^-^² s^-^¹) were measured using a LI-600N Porometer/Fluorometer. Physiological measurements were taken after 30–60 and 90 days. At the end of the experiment (day 90), gas exchange parameters, including intracellular CO_2_ concentration (C_i_, µmol mol^-^¹), stomatal conductance (*g_s_*, mol m^-2^ s^-1^), net CO_2_ assimilation rate (A, µmol m^-^²s^-^¹), transpiration rate (E, mmol m^-^²s^-^¹), and photosynthetic water use efficiency (WUE, mmol mol^-^¹) were measured using a CIRAS-4 Portable Photosynthesis System (IRGA, infrared gas analyzer), in L. *japoniucm* ‘Texanum’ and *L.vulgare*. No results were obtained from *L. sinense*, although the plants in treatment W2 were alive, we were unable to measure the data due to technical limitations of the instrument.

### 2.7 Statistical analysis

All analyzed data were checked for the normality of variance by using Shapiro–Wilks test (*p* > 0.05). For all parameters, mean differences were computed using a one-way and univariate ANOVA with Tukey’s *post-hoc* test (*p* ≤ 0.05) and significant differences among two means were compared using Student’s t-test (*p* ≤ 0.05). Analyses were performed using IBM SPSS Statistics version 29.0.1.0 software. When insufficient biological replicates (fewer than three) were available for a given treatment, it was excluded from statistical analysis and not reported in the results A principal component analysis (PCA) biplot was performed using PAST 5.3 software, including morpho-physiological and vegetation index results.

## 3 Results

In general, at the end of the experiment, no species survived treatments W1, S1W1, and S2W1, as shown in **Supplementary Table S1**. *L. sinense* survived only to treatment W2, *L. japonicum* ‘Texanum’ survived treatments C, S1, S2, W2, and S1W2, while *L. vulgare* survived treatments C, S1, S2, W2, S1W2, and S2W2.

### 3.1 Morphological measurements by PhenoPlant

At the midpoint of the experiment, the species × treatment interaction was significant for the 3D leaf area, digital biomass, and plant height (**Table 3**). In *L. sinense*, W2 showed a reduction of approximately 85% in 3D leaf area and digital biomass compared to the control, while plant height was not significantly affected. In *L. japonicum* ‘Texanum’, treatments S1, S2, W2, and S1W2 significantly reduced 3D leaf area and digital biomass compared with the control, with no significant differences among treatments. Relative to the control, S1 reduced 3D leaf area and digital biomass by 38% and 36%, respectively; S2 by 41% and 39%; W2 by 44% and 48%; and S1W2 by 50% and 48%. Treatments didn’t affect plant height. In *L. vulgare*, S1W2 and S2W2 combinations showed significantly lower 3D leaf area and digital biomass than W2. Specifically, S1W2 reduced the 3D leaf area and digital biomass by 46% and 56%, respectively, while S2W2 caused reductions of 44% and 65% compared with W2. In contrast, W2 values were comparable to the control. At the end of the experiment, a significant species × treatment interaction was detected for 3D leaf area and digital biomass. In *L. sinense*, although the mortality rate remained constant in W2, 3D leaf area and digital biomass increased by 99% and 84%, respectively, relative to the midpoint values, but remained significantly lower than the control (80% and 84%, respectively). In *L. japonicum* ‘Texanum’, 3D leaf area did not differ among S1, S2, W2, and S1W2, but was significantly higher in C. However, all treatments showed significantly lower 3D leaf area than the control, whereas digital biomass under S2 remained comparable to the control, with a 34% increase relative to midpoint measurements. In *L. vulgare*, 3D leaf area was significantly lower in treatments S1 (6516 mm²), S2 (7986 mm²), S1W2 (4798 mm²), and S2W2 (2706 mm²) compared to C (15129 mm²). W2 (13731 mm²) was not different from S2, and C. Compared with midpoint measurements, W2 exhibited a 2.48% increase in 3D leaf area, despite an unchanged mortality rate. The W2 treatment also showed significantly higher digital biomass values (5204311 mm³) than S1 (1814277 mm³), S1W2 (1558531 mm³), and S2W2 (948587 mm³), but did not differ significantly from S2 or the control (**Table 4**).

**Table 3:** Significant species × treatment interaction in *Ligustrum sinense*, *Ligustrum japonicum* ‘Texanum, and *Ligustrum vulgare* for morphological traits measured at midpoint of experiment: 3D leaf area in mm², digital biomass in mm³, and mean plant height in mm.

Treatments	*Ligustrum sinense*			*Ligustrum japonicum* ‘Texanum’			*Ligustrum vulgare*		
	3D Leaf area (mm^2^)	Digital biomass (mm^3^)	Plant height averaged (mm)	3D Leaf area (mm^2^)	Digital biomass (mm^3^)	Plant height averaged (mm)	3D Leaf area (mm^2^)	Digital biomass (mm^3^)	Plant height averaged (mm)
C	22633	5032478	219	20104 ^a^	3613119 ^a^	179	16888 ^a^	7118538 ^a^	413 ^a^
S1	/^§^	/^§^	/^§^	12537 ^b^	2326626 ^b^	191	9899 ^b,c^	2903601 ^b,c^	309 ^a,b^
S2	/^§^	/^§^	/^§^	11888 ^b^	2212688 ^b^	181	10477 ^b,c^	3636646 ^b,c^	334 ^a,b^
W2	3782	752245 ^b^	197	11274 ^b^	1877115 ^b^	170	13399 ^a,b^	5281804 ^a,b^	373 ^a^
S1W2	/^§^	/^§^	/^§^	9951 ^b^	1847477 ^b^	185	7175 ^c^	2334563 ^c^	302 ^a,b^
S2W2	/^§^	/^§^	/^§^	/^§^	/^§^	/^§^	7495 ^c^	1865828 ^c^	240 ^b^
*Sig.*	***	***	ns	***	***	ns	***	***	**

The treatments included C (control, 90% pot capacity); S1 (150 mM NaCl); S2 (300 mM NaCl); W2 (60% pot capacity); S1W2 (150 mM NaCl + water stress at 60% pot capacity); and S2W2 (300 mM NaCl + water stress at 60% pot capacity). Different letters indicate significant differences (*p* < 0.05) among treatments within each species. ns, not significant; **p < 0.01; ***p < 0.001 represents the significance of main effects from ANOVA. The values in the column followed by the same letter are not significantly different (Tukey’s test). ^§^Data excluded for insufficient biological replicates.

**Table 4:** Significant species × treatment interaction in *Ligustrum sinense*, *Ligustrum japonicum* ‘Texanum’, and *Ligustrum vulgare* for morphological traits measured at the end of the experiment: 3D Leaf Area (mm²) and Digital Biomass (mm³), measured at the end of the experiment.

Treatments	*Ligustrum sinense*		*Ligustrum japonicum* ‘Texanum’		*Ligustrum vulgare*	
	3D Leaf area (mm^2^)	Digital biomass (mm^3^)	3D Leaf area (mm^2^)	Digital biomass (mm^3^)	3D Leaf area (mm^2^)	Digital biomass (mm^3^)
C	37194	8717853	26509 ^a^	4870814 ^a^	15129 ^a^	6066283 ^a^
S1	/^§^	/^§^	11390 ^b^	2245759 ^b^	6516 ^c^	1814277 ^b^
S2	/^§^	/^§^	10640 ^b^	2972484 ^a,b^	7986 ^b,c^	3507468 ^a,b^
W2	7525	1389993	11019 ^b^	1844893 ^b^	13731 ^a,b^	5204311 ^a^
S1W2	/^§^	/^§^	6591 ^b^	1189787 ^b^	4798 ^c^	1558531 ^b^
S2W2	/^§^	/^§^	/^§^	/^§^	2706 ^c^	948587 ^b^
*Sig.*	***	***	***	***	***	***

The treatments included C (control, 90% pot capacity); S1 (150 mM NaCl); S2 (300 mM NaCl); W2 (60%pot capacity); S1W2 (150 mM NaCl + 60% pot capacity); and S2W2 (300 mM NaCl + 60% pot capacity). Different letters indicate significant differences (*p* < 0.05) among treatments within each species. ns, not significant; ***p < 0.001 represents the significance of main effects from ANOVA. The values in the column followed by the same letter are not significantly different (Tukey’s test). ^§^Data excluded for insufficient biological replicates.

### 3.2 Vegetation index by Phenoplant

At the midpoint of the experiment, the species × treatment interaction was not significant for the vegetation indices GLI, NDVI, NPCI, and PSRI. By the end of the experiment, however, the interaction was significant for NDVI, NPCI, and PSRI (**Table 5**). In *L. sinense*, W2 showed a significant reduction in NDVI compared with the control, suggesting reduced canopy vigor and chlorophyll content consistent with plant stress. Similarly, the higher NPCI observed in W2 relative to the control (−0.041 vs. −0.080) indicates a potential physiological stress associated with reduced chlorophyll levels. In *L. japonicum* ‘Texanum’, NDVI and PSRI did not differ significantly among treatments. NPCI value in S1 (0.017) differed significantly from the S1W2 combination (0.185), which could indicate more stress in S1W2, but non different from S2 and W2. In *L. vulgare*, the treatments S1 (0.527), S2 (0.488), and S2W2 (0.463) showed significantly lower NDVI values than W2 (0.674) with a reduction in canopy vigor. W2 did not differ significantly from S1W2 (0.555) and the control (0.589). The S2W2 (0.273) showed significantly higher NPCI values than C (0.089), W2 (0.023), and S1W2 (0.129), suggesting a decline in chlorophyll content. S2W2 did not differ from treatments S1 (0.157) and S2 (0.205). Treatments C, W2, and S1W2 showed the lowest NPCI values, indicative of a better physiological state. Finally, treatments S2 and S2W2 differed significantly from W2, showing higher PSRI values and indicating clear leaf senescence. Treatments C, S1, and S1W2 had intermediate PSRI values, not significantly different from either S2 and S2W2 or W2 (**Table 5**).

**Table 5:** Significant species × treatment interaction in *Ligustrum sinense*, *Ligustrum japonicum* ‘Texanum’, and *Ligustrum vulgare* for vegetation indices measured with the PhenoPlant at the end of the experiment.

Treatments	*Ligustrum sinense*			*Ligustrum japonicum* ‘Texanum’			*Ligustrum vulgare*		
	NDVI	NPCI	PSRI	NDVI	NPCI	PSRI	NDVI	NPCI	PSRI
C	0.692	-0.080	-0.001	0.705	0.081 ^a,b^	0.032	0.589 ^a,b^	0.089 ^b,c^	0.089 ^a,b^
S1	/^§^	/^§^	/^§^	0.686	0.017 ^b^	0.033	0.527 ^b^	0.157 ^a,b^	0.106 ^a,b^
S2	/^§^	/^§^	/^§^	0.676	0.065 ^a,b^	0.044	0.488 ^b^	0.205 ^a,b^	0.131 ^a^
W2	0.645	-0.041	0.017	0.691	0.065 ^a,b^	0.030	0.674 ^a^	0.023 ^b,c^	0.016 ^b^
S1W2	/^§^	/^§^	/^§^	0.633	0.185 ^a^	0.078	0.555 ^a,b^	0.129 ^b,c^	0.075 ^a,b^
S2W2	/^§^	/^§^	/^§^	/^§^	/^§^	/^§^	0.463 ^b^	0.273 ^a^	0.170 ^a^
*Sig.*	*	*	ns	ns	*	ns	***	***	**

The vegetation indices include Normalized Difference Vegetation Index (NDVI),Normalized Pigment Chlorophyll Index (NPCI), and Plant Senescence Reflectance Index (PSRI). The treatments included C (control, 90% pot capacity); S1 (150 mM NaCl); S2 (300 mM NaCl); W2 (60% pot capacity); S1W2 (150 mM NaCl + 60% pot capacity); and S2W2 (300 mM NaCl + 60% pot capacity). Different letters indicate significant differences (*p* < 0.05) among treatments within each species. ns, not significant; *p < 0.05; **p < 0.01; ***p < 0.001 represents the significance of main effects from ANOVA. The values in the column followed by the same letter are not significantly different (Tukey’s test). ^§^Data excluded for insufficient biological replicates.

### 3.3 Vegetation index by Spectroradiometer

After 60 days, the species × treatment interaction was significant for WBI and NDWI. Spectroradiometer measurements in *L. sinense* showed a slight reduction in WBI under W2 compared with the control, suggesting lower leaf water content, whereas NDWI value increased slightly in W2 realtive to the control. In *L. japonicum* ‘Texanum’, S2 and S1W2 exhibited higher NDWI but lower WBI values than S1, W2, and the control, suggesting reduced leaf water content without evidence of severe stress. S2 and W2 did not differ significantly in WBI and NDWI values. In L*.vulgare*, W2 showed significantly higher WBI values than S1, S2, S1W2, and S2W2, but did not differ from the control, while NDWI values did not differ among treatments (**Table 6**). At the end of the treatments, the species × treatment interaction remained significant for WBI and NDWI. No significant differences in WBI or NDWI were detected in *L. sinense*. In *L. japonicum* ‘Texanum’, NDWI values in S1, S2, and S1W2 did not differ from each other but were significantly higher than those in the control and W2, which were similar. WBI was significantly higher in W2, and control compared to S2 and S1W2, whereas S1 showed an intermediate value not different from other treatments. In *L. vulgare*, only WBI differed significantly among treatments. W2 exhibited a higher WBI value than S1, while the remaining treatments did not differ from either S1 and W2, nor form each other (**Table 7**).

**Table 6:** Significant species × treatment interaction in *Ligustrum sinense*, *Ligustrum japonicum* ‘Texanum’, and *Ligustrum vulgare* for Water Band Index (WBI) and Normalized Difference Water Index (NDWI), measured 60 days after the start of the experiment.

Treatments	*Ligustrum sinense*		*Ligustrum japonicum* ‘Texanum’		*Ligustrum vulgare*	
	WBI	NDWI	WBI	NDWI	WBI	NDWI
C	0.997	0.032	0.967 ^a^	0.074 ^c^	0.979 ^a,b^	0.057
S1	/^§^	/^§^	0.950 ^b^	0.092 ^b^	0.973 ^b^	0.057
S2	/^§^	/^§^	0.931 ^c^	0.115 ^a^	0.974 ^b^	0.065
W2	0.986	0.037	0.955 ^a,b^	0.082 ^b,c^	0.988 ^a^	0.050
S1W2	/^§^	/	0.931 ^c^	0.110 ^a^	0.972 ^b^	0.055
S2W2	/^§^	/^§^	/^§^	/^§^	0.972 ^b^	0.057
*Sig*.	**	*	***	***	**	ns

The treatments included C (control, 90% pot capacity); S1 (150 mM NaCl); S2 (300 mM NaCl); W2 (60% pot capacity); S1W2 (150 mM NaCl + 60% pot capacity); and S2W2 (300 mM NaCl + 60% pot capacity). Different letters indicate significant differences (*p* < 0.05) among treatments within each species. ns, not significant; *p < 0.05; **p < 0.01; ***p < 0.001 represents the significance of main effects from ANOVA. The values in the column followed by the same letter are not significantly different (Tukey’s test). ^§^Data excluded for insufficient biological replicates.

**Table 7:** Significant species × treatment interaction in *Ligustrum sinense*, *Ligustrum japonicum* ‘Texanum’, and *Ligustrum vulgare* for Water Band Index (WBI) and Normalized Difference Water Index (NDWI), measured 90 days after the start of the experiment.

Treatments	*Ligustrum sinense*		*Ligustrum japonicum* ‘Texanum’		*Ligustrum vulgare*	
	WBI	NDWI	WBI	NDWI	WBI	NDWI
C	0.988	0.043	0.958 ^a^	0.080 ^b^	0.970 ^a,b^	0.050
S1	/^§^	/^§^	0.940 ^a,b^	0.102 ^a,b^	0.965 ^b^	0.059
S2	/^§^	/^§^	0.929 ^b^	0.120 ^a^	0.975 ^a,b^	0.058
W2	0.982	0.038	0.953 ^a^	0.080 ^b^	0.980 ^a^	0.052
S1W2	/^§^	/^§^	0.921 ^b^	0.112 ^a,b^	0.979 ^a,b^	0.051
S2W2	/^§^	/^§^	/^§^	/^§^	0.970 ^a,b^	0.054
*Sig*.	ns	ns	***	*	*	ns

The treatments included C (control, 90% pot capacity); S1 (150 mM NaCl); S2 (300 mM NaCl); W2 (60% pot capacity); S1W2 (150 mM NaCl + 60% pot capacity); S2W2 (300 mM NaCl + 60% pot capacity). Different letters indicate significant differences (*p* < 0.05) among treatments within each species. ns, not significant; *p < 0.05; ***p < 0.001 represents the significance of main effects from ANOVA. The values in the column followed by the same letter are not significantly different (Tukey’s test). ^§^Data excluded for insufficient biological replicates.

### 3.4 Shoot and root dry biomass, and root index

**Figure 2:**
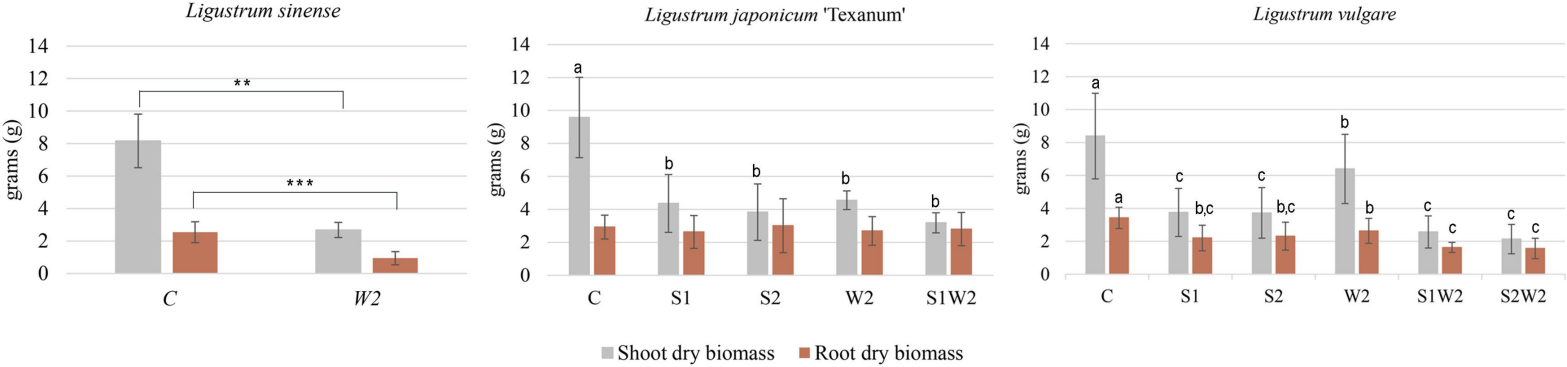
Histograms illustrate the significant species × treatment interaction for shoot and root dry biomass in *Ligustrum sinense*, *Ligustrum japonicum* ‘Texanum’, and *Ligustrum vulgare*. Treatments included: control (C), salt stress at 150 mM NaCl (S1), salt stress at 300 mM NaCl (S2), water stress at 60% pot capacity (W2), 150 mM NaCl and 60% pot capacity (S1W2), and 300 mM NaCl and 60% pot capacity (S2W2). Different letters indicate statistically significant differences among species and treatments (Tukey’s test, *p* < 0.05). no letters = not significant; **p < 0.01; ***p < 0.001 denote significance levels of main effects according to ANOVA.

**Figure 3:**
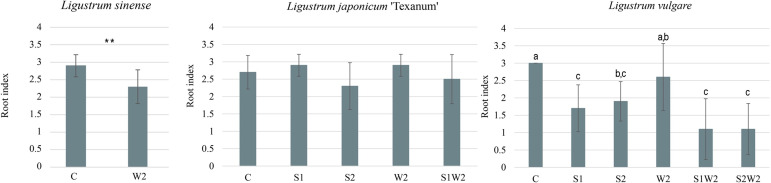
Histograms illustrate the significant species × treatment interaction for root index in *Ligustrum sinense, Ligustrum japonicum* ‘Texanum’, and *Ligustrum vulgare*. Treatments included: control (C), salt stress at 150 mM NaCl (S1), salt stress at 300 mM NaCl (S2), water stress at 60% pot capacity (W2), 150 mM NaCl and 60% pot capacity (S1W2), and 300 mM NaCl and 60% pot capacity (S2W2). Different letters indicate statistically significant differences among species and treatments (Tukey’s test, *p* < 0.05). no letters, not significant; **p < 0.01 denote significance levels of main effects according to ANOVA.

After 90 days, the interaction between species × treatment was significant for dry shoot and root biomass, and root index. In *L. sinense*, the W2 treatment resulted in a 68% reduction in shoot dry biomass compared to C (2.68 g vs 8.16 g) and a 62% reduction in root dry biomass (0.94 g vs 2.5 g). In *L. japonicum* ‘Texanum’, the shoot dry biomass did not differ significantly among the stress treatments S1, S2, W2, and S1W2, but the value in C was significantly higher (9.5 g). The treatments didn’t differ in root dry biomass. In *L. vulgare* the W2 treatment showed a significant reduction of 24% in shoot dry biomass compared to C (6.39 g and 8.39 g), but values in W2 were significantly higher than those observed under S1, S2, S1W2, and S2W2. For root dry biomass, the combined treatments S1W2 (1.6 g) and S2W2 (1.6 g) resulted in significantly lower values than W2 (2.6 g) but didn’t differ from S1 and S2 (**Figure 2**). In *L. sinense*, the root index showed significantly lower root development in the W2 (2.3) stress treatment compared to the control (2.9). *In L. japonicum* ‘Texanum’, the root index showed no significant differences between treatments. In *L. vulgare*, the W2 (2.6) treatment did not differ significantly from C, but was significantly higher than S1 (1.7), S1W2 (1.1), and S2W2 (1.1). The S1, S2, S1W2, and S2W2 treatments showed no significant differences between them (**Figure 3**).

### 3.5 Physiological measurements

Physiological measurements were also collected 30 days after the start of treatment. At this point, the species × treatment interaction was significant for SPAD, g_s_, E, and ETR (**Table 8**). In *L. sinense*, chlorophyll content was significantly higher in W2 (65.87 SPAD units) than in C (49.99), S1 (52.47), and S2 (43.53), which did not differ significantly from each other. No significant differences in g_s_, E, and ETR were detected among C, S1, and S2. In *L. japonicum* ‘Texanum’, severe salt stress (S2) resulted in a significant increase in chlorophyll content (SPAD unit 60.68) although it did not differ significantly from S1 (55.85). The lowest chlorophyll values were observed in S2W1, S1W2, and W2. Treatment S2 also showed the most marked stomatal conductance (g_s_ 0.076 mol m^-2^ s^-1^) and transpiration rate (E 1.45 mmol m^-2^ s^-1^) among treatments, with a marked reduction in ETR value (17.7 µmol e^-^ m^-^² s^-^¹). Although this represented the lowest ETR value, it did not differ significantly from S1 or S2W1. In contrast, W2 highlighted increased photosynthetic electron transport activity, with the highest value (ETR 74.6 µmol e^-^ m^-^² s^-^¹), differing significantly from S1 (26.83 µmol e^-^ m^-^² s^-^¹), S2 (17.73 µmol e^-^ m^-^² s^-^¹), and S2W1 (23.85 µmol e^-^ m^-^² s^-^¹). ETR values in the control (54.97 µmol e^-^ m^-^² s^-^¹) and S1W2 (55.65 µmol e^-^ m^-^² s^-^¹) were intermediate and did not differ significantly from W2. In *Ligustrum vulgare*, W2 showed the highest chlorophyll content (SPAD unit 44.03). The lowest values were presented in S2W1 (26.71), which did not differ significantly from S2W2 (27.08), S1W2 (32.34), and S2 (32.41). Stomatal conductance and transpiration were highest in the control, as expected, but did not differ from treatments. High salt concentration caused a strong reduction in photosynthetic electron transport activity (3.1 µmol e^-^ m^-^² s^-^¹) In contrast, S1 (84.20 µmol e^-^ m^-^² s^-^¹), S1W1(70.05 µmol e^-^ m^-^² s^-^¹), and S1W2 (83.91µmol e^-^ m^-^² s^-^¹) showed higher ETR values; these did not differ significantly from W2, S2W1, S2W2 and C. After 60 days, only SPAD and ETR showed a significant species × treatment interaction. In *L. sinense*, W2 resulted in a significant reduction in chlorophyll content (SPAD unit 54.12) compared with the control (66.48), whereas electron transport activity did not differ among treatments. In *L. japonicum* ‘Texanum’, only SPAD differed significantly among treatments. W2 showed higher chlorophyll content (53.33 SPAD units) than S2 (42.04), but did not differ from S1 (48.07), and S1W2 (46.09). In *L. vulgare*, W2 exhibited the highest chlorophyll content (SPAD unit 61.26), while S2, S1W2, and S2W2 showed the lowest values. For ETR, the lowest values were recorded in S2W2 (42.55 µmol e^-^ m^-^² s^-^¹) and S2 (42.84 µmol e^-^ m^-^² s^-^¹) compared to S1W2 (81.94 µmol e^-^ m^-^² s^-^¹) and W2 (74.75 µmol e^-^ m^-^² s^-^¹) (**Table 9**). At the end of the experiment, the species × treatment interaction was significant for SPAD, g_s_, E, and VPD. In *L. sinense*, W2 reported lower chlorophyll content, g_s,_ transpiration and VPD compared to C. In *L. japonicum* ‘Texanum’, moderate water stress W2 showed the highest quantity of chlorophyll (SPAD units 61.44) than its combination with salt, S1W2 (44.04). Stomatal conductance and transpiration were significantly affected by treatments. S2 exhibited high values of g_s_ and E, indicating pronounced stomatal opening and elevated water flux. W2 also showed increased gas exchange, not different from S2, but no elevated transpiration rate. In contrast, C, S1, and S1W2 maintained low gs and E value, reflecting a more conservative water use strategy. VPD was not different from treatments. In *L. vulgare*, treatments significantly affected chlorophyll content, stomatal conductance, transpiration, and VPD. Plants under moderate drought stress W2 maintained the highest value of chlorophyll content (63.03 SPAD units), whereas severe salt stress treatments (S2 and S2W2) showed a reduction of chlorophyll. Stomatal conductance was highest in S1 (0.167 mol m^-2^ s^-1^), W2 (0.140 mol m^-2^ s^-1^), and S1W2 (0.160 mol m^-2^ s^-1^), not different from the control (0.199 mol m^-^² s^-^¹). In contrast, S2 showed the lowest g_s_ value (0.021 mol m^-^² s^-^¹) compared to moderate salt stress S1 and C. Transpiration rate was significantly reduced in S2 (0.42 mmol m^-^² s^-^¹) than C and S1W2. Leaf VPD was significantly higher in W2 and S1W2 (2.23 and 2.22 kPa, respectively) than in the control and S1. Treatments S2 and S2W2 showed intermediate VPD values that did not differ from either group (**Table 10**). IRGA parameters did not show statistically significant differences in *L. japoniucm* ‘Texanum’, whereas A, WUE, and C_i_ were significantly different in *L. vulgare*. Treatments S1 and S2 showed severe stress conditions, as indicated by negative net CO_2_ assimilation (−0.43 and −0.57 µmol m^-^² s^-^¹, respectively) and negative water use efficiency (−0.71 and −0.27 mmol mol^-^¹). The higher intercellular CO_2_ concentration observed under these treatments suggests that CO_2_ diffusion into the leaf was not limiting; however, carbon was not effectively assimilated, resulting in water loss without carbon gain. The W2 treatment showed good photosynthetic performance with significantly higher net CO_2_ assimilation than all other treatments (A = 2.33 µmol m^-^² s^-^¹). The WUE value was positive and high, while C_i_ value was lower than in the S1 and S2 treatments, but not different from S1W2 and S2W2 (**Table 11**).

**Table 8:** Significant species × treatment interaction in *Ligustrum sinense*, *Ligustrum japonicum* ‘Texanum’, and *Ligustrum vulgare* for SPAD (units), stomatal conductance (g_s_, mol m^-^² s^-^¹), transpiration rate (E, mmol m^-^² s^-^¹),and electron transport rate (ETR, μmol electrons m^-^² s^-^¹), measured 30 days after the start of the experiment.

*Ligustrum sinense*				
Treatments	SPAD	g_s_ mol m^-2^ s^-1^	E mmol m^-2^ s^-1^	ETR µmol e^-^ m^-^² s^-^¹
C	49.99 ^b^	0.009	0.24	60.31
S1	52.47 ^b^	0.011	0.30	46.11
W2	65.87 ^a^	0.006	0.16	47.00
S1W2	43.53 ^b^	0.008	0.20	60.92
*Sig.*	***	ns	ns	ns

*Ligustrum japonicum* ‘Texanum’				
Treatments	SPAD	g_s_ mol m^-2^ s^-1^	E mmol m^-2^ s^-1^	ETR µmol e^-^ m^-^² s^-^¹
C	46.47 ^b,c^	0.006 ^b^	0.14 ^b^	54.97 ^a,b^
S1	55.85 ^a,b^	0.005 ^b^	0.12 ^b^	26.83 ^b,c^
S2	60.68 ^a^	0.076 ^a^	1.45 ^a^	17.73 ^c^
W2	42.22 ^b,c^	0.007 ^b^	0.15 ^b^	74.59 ^a^
S1W2	41.66 ^b,c^	0.037 ^b^	0.75 ^b^	55.65 ^a,b^
S2W1	40.42 ^c^	0.026 ^b^	0.57 ^b^	23.85 ^b,c^
*Sig.*	***	***	***	***

*Ligustrum vulgare*				
Treatments	SPAD	g_s_ mol m^-2^ s^-1^	E mmol m^-2^ s^-1^	ETR µmol e^-^ m^-^² s^-^¹
C	46.00 ^a^	0.082 ^a^	1.43 ^a^	30.67 ^a,b^
S1	37.07 ^b^	0.017 ^b^	0.42 ^b^	84.20 ^a^
S2	32.41 ^b,c,d^	0.016 ^b^	0.37 ^b^	3.06 ^c^
W2	44.03 ^a^	0.019 ^b^	0.47 ^b^	35.83 ^a,b^
S1W1	34.74 ^b,c^	0.009 ^b^	0.21 ^b^	70.05 ^a^
S1W2	32.34 ^b,c,d^	0.007 ^b^	0.17 ^b^	83.91 ^a^
S2W1	26.71 ^d^	0.004 ^b^	0.09 ^b^	55.08 ^a,b^
S2W2	27.08 ^c,d^	0.008 ^b^	0.15 ^b^	46.60 ^a,b^
*Sig.*	***	***	***	***

The treatments included C (control, 90% pot capacity); S1 (150 mM NaCl); S2 (300 mM NaCl); W1 (30% the pot capacity); W2 (60% pot capacity); S1W1 (150 mM NaCl + 30% pot capacity); S1W2 (150 mM NaCl + 60 pot capacity); S2W1 (150 mM NaCl + 30% pot capacity); and S2W2 (300 mM NaCl + 60% pot capacity). Different letters indicate significant differences (*p* < 0.05) among treatments within each species. ns, not significant; ***p < 0.001 represents the significance of main effects from ANOVA. The values in the column followed by the same letter are not significantly different (Tukey’s test). ^§^Data excluded for insufficient biological replicates.

**Table 9:** Significant species × treatment interaction in *Ligustrum sinense*, *Ligustrum japonicum* ‘Texanum’, and *Ligustrum vulgare* for SPAD (units) and electron transport rate (ETR, μmol electrons m^-^² s^-^¹), measured 60 days after the start of the experiment.

Treatments	*Ligustrum sinense*		*Ligustrum japonicum* ‘Texanum’		*Ligustrum vulgare*	
	SPAD	ETR µmol e^-^ m^-^² s^-^¹	SPAD	ETR µmol e^-^ m^-^² s^-^¹	SPAD	ETR µmol e^-^ m^-^² s^-^¹
C	66.48	62.94	63.61 ^a^	64.75	55.62 ^a^	76.79 ^a,b^
S1	/^§^	/^§^	48.07 ^b,c^	69.45	42.20 ^b^	44.79 ^b,c^
S2	/^§^	/^§^	42.04 ^c^	47.48	26.68 ^c^	42.84 ^c^
W2	54.12	78.65	55.33 ^a,b^	58.29	61.26 ^a^	74.75 ^a,b^
S1W2	/^§^	^§^	46.09 ^b,c^	51.92	30.82 ^c^	81.94 ^a^
S2W2	/^§^	/^§^	/^§^	/^§^	29.65 ^c^	42.55 ^c^
*Sig*.	***	ns	***	ns	***	**

The treatments included C (control, 90% pot capacity); S1 (150 mM NaCl); S2 (300 mM NaCl); W2 (60% pot capacity); S1W2 (150 mM NaCl + 60% pot capacity); and S2W2 (300 mM NaCl + 60% pot capacity). Different letters indicate significant differences (p < 0.05) among treatments within each species. ns=not significant; **p < 0.01; ***p < 0.001 represents the significance of main effects from ANOVA. The values in the column followed by the same letter are not significantly different (Tukey’s test). ^§^Data excluded for insufficient biological replicates.

**Table 10:** Significant species × treatment interaction in *Ligustrum sinense*, *Ligustrum japonicum* ‘Texanum’, and *Ligustrum vulgare* for SPAD (units), stomatal conductance (g_s_, mol m^-^² s^-^¹), transpiration rate (E, mmol m^-^² s^-^¹), and leaf vapor pressure deficit (VPD Leaf, kPa), measured 90 days after the start of the experiment.

*Ligustrum sinense*				
Treatments	SPAD	g_s_ mol m^-2^ s^-1^	E mmol m^-2^ s^-1^	VPD Leaf kPa
C	62.53	0.033	0.78	2.28
W2	55.96	0.005	0.09	1.76
*Sig.*	*	*	**	***

*Ligustrum japonicum* ‘Texanum’				
Treatments	SPAD	g_s_ mol m^-2^ s^-1^	E mmol m^-2^ s^-1^	VPD Leaf kPa
C	62.65 ^a^	0.025 ^b^	0.59 ^c^	2.12
S1	52.13 ^a,b^	0.035 ^b^	0.78 ^c^	1.98
S2	53.05 ^a,b^	0.223 ^a^	5.19 ^a^	2.35
W2	61.44 ^a^	0.170 ^a^	3.35 ^b^	1.81
S1W2	44.04 ^b^	0.043 ^b^	0.66 ^c^	1.60
*Sig.*	***	***	***	ns

*Ligustrum vulgare*				
Treatments	SPAD	g_s_ mol m^-2^ s^-1^	E mmol m^-2^ s^-1^	VPD Leaf kPa
C	61.19 ^a^	0.199 ^a^	3.37 ^a^	1.48 ^b^
S1	36.46 ^b^	0.167 ^a,b^	2.90 ^a,b^	1.73 ^b^
S2	26.55 ^b,c^	0.021 ^c^	0.42 ^b^	1.86 ^a,b^
W2	63.03 ^a^	0.140 ^a,b,c^	3.00 ^a,b^	2.23 ^a^
S1W2	34.89 ^b^	0.160 ^a,b,c^	3.49 ^a^	2.22 ^a^
S2W2	22.56 ^c^	0.027 ^b,c^	0.50 ^b^	1.76 ^a,b^
*Sig.*	***	*	*	***

The treatments included C (control, 90% pot capacity); S1 (150 mM NaCl); S2 (300 mM NaCl); W2 (60% pot capacity); S1W2 (150 mM NaCl + 60% pot capacity); and S2W2 (300 mM NaCl + 60% pot capacity). Different letters indicate significant differences (p < 0.05) among treatments within each species. ns = not significant; *p < 0.05; **p < 0.01; ***p < 0.001 represents the significance of main effects from ANOVA. The values in the column followed by the same letter are not significantly different (Tukey’s test). ^§^Data excluded for insufficient biological replicates.

**Table 11:** Effects of treatments on net CO_2_ assimilation (A, mmol m-² s-¹), stomatal conductance (g_s_, mol m-² s-¹), transpiration rate (E, mmol m-² s-¹), water use efficiency (WUE, mmol mol-¹), and intracellular CO_2_ concentration (C_i_, μmol·mol⁻¹), in *L. japonicum* 'Texanum' and *L. vulgare*, measured 90 days after the start of the experiment.

*Ligustrum japonicum* ‘Texanum’					
Treatments	A μmol·m^-2^·s-^1^	g_s_ mol m^-2^ s^-1^	E mmol m^-2^ s^-1^	WUE mmol·mol^-1^	C_i_ μmol·mol^-1^
C	0.49	0.041	0.64	0.78	418.26
S1	0.50	0.037	0.55	0.95	416.13
S2	0.44	0.046	0.67	0.60	424.58
W2	0.34	0.036	0.55	0.62	423.31
S1W2	0.38	0.040	0.59	0.63	423.26
*Sig.*	ns	ns	ns	ns	ns

*Ligustrum vulgare*					
Treatments	A μmol·m^-2^·s-^1^	g_s_ mol m^-2^ s^-1^	E mmol m^-2^ s^-1^	WUE mmol·mol^-1^	C_i_ μmol·mol^-1^
C	1.37 ^a,b^	0.055	0.75	1.88 ^a^	397.79 ^b^
S1	-0.43 ^d^	0.047	0.60	-0.71 ^c^	455.53 ^a^
S2	-0.57 ^c,d^	0.063	0.95	-0.27 ^c^	443.53 ^a^
W2	2.33 ^a^	0.109	1.71	1.41 ^a,b^	397.41 ^b^
S1W2	0.86 ^b,c^	0.082	1.30	0.67 ^a,b,c^	418.48 ^a,b^
S2W2	0.17 ^c,d^	0.078	1.26	0.25 ^b,c^	428.22 ^a,b^
*Sig.*	***	ns	ns	**	**

Treatments included C (control, 90% pot capacity); S1 (150 mM NaCl); S2 (300 mM NaCl); W2 (60% pot capacity); S1W2 (150 mM NaCl + 60% pot capacity); and S2W2 (300 mM NaCl + 60%pot capacity). Different letters indicate significant differences (p < 0.05) among treatments within each species. ns, not significant; **p < 0.01; ***p < 0.001 represents the significance of main effects from ANOVA. The values in the column followed by the same letter are not significantly different (Tukey’s test).

### 3.6 Principal component analysis

**Figure 4:**
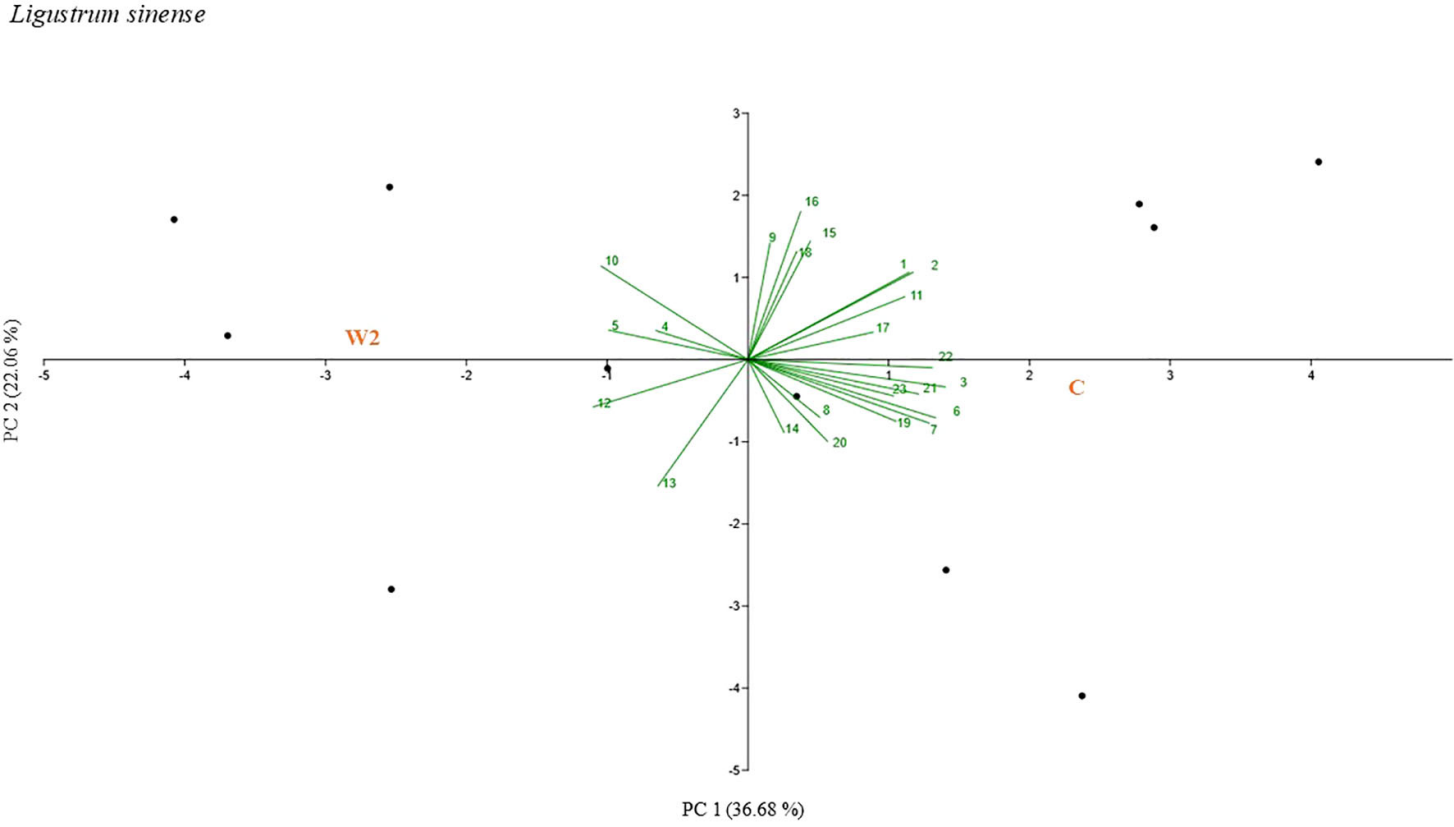
Principal component analysis (PCA)-biplot performed for g_s_ (1), E (2), VPD (3), PhiPS2 (4), ETR (5), 3D leaf area (6), digital biomass (7), plant heigh average (8), SPAD (9), GLI (10), NDVI(11), NPCI (12), PSRI (13), SR (14), EVI (15), SAVI (16), PRI (17), MASVI2 (18), WBI (19), NDWI (20), dry shoot biomass (21), dry root biomass (22), root index (23) values of *Ligustrum sinense*. The labels in red capital letters represent the centroids of the sample clouds for each treatment represented by C and W2.

**Figure 5:**
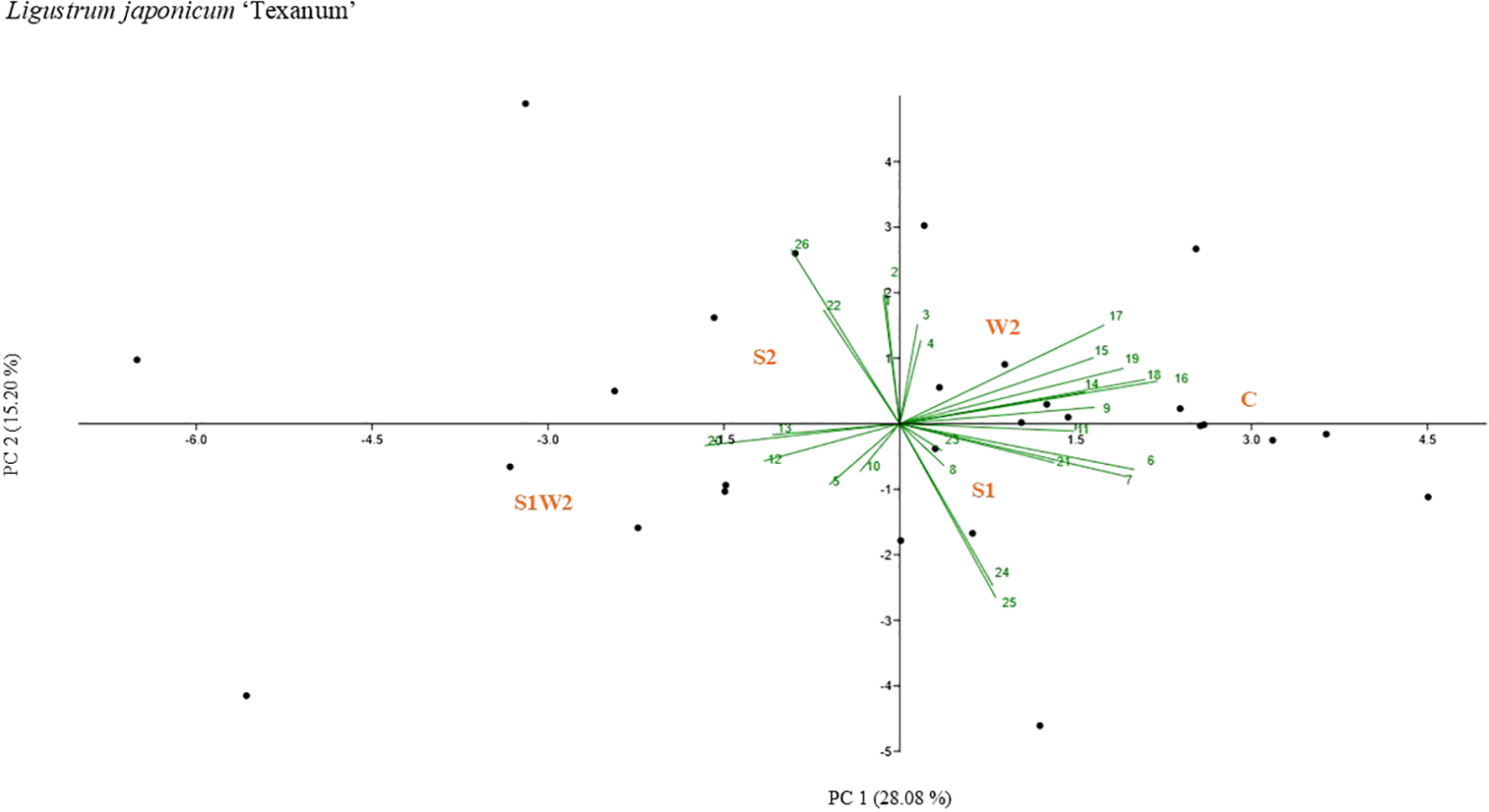
Principal component analysis (PCA)-biplot performed for g_s_ (1), E (2), VPD (3), PhiPS2 (4), ETR (5), 3D leaf area (6), digital biomass (7), plant heigh average (8), SPAD (9), GLI (10), NDVI(11), NPCI (12), PSRI (13), SR (14), EVI (15), SAVI (16), PRI (17), MASVI2 (18), WBI (19), NDWI (20), dry shoot biomass (21), dry root biomass (22), root index (23), A (24), WUE (25), C_i_ (26) values of *Ligustrum japonicum* ‘Texanum’. The labels in red capital letters represent the centroids of the sample clouds for each treatment represented by C, S1, S2, W2, and S1W2.

**Figure 6:**
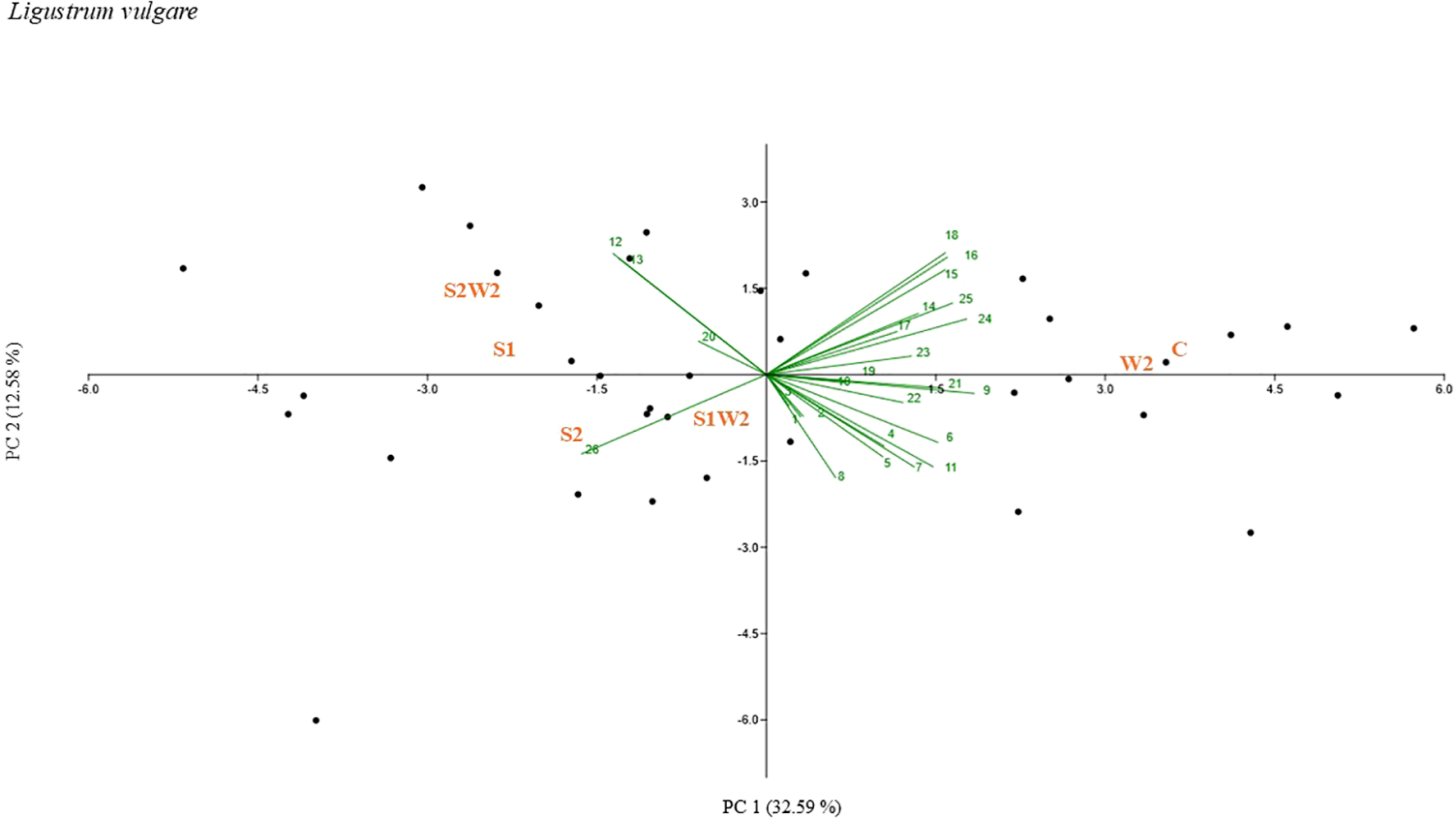
Principal component analysis (PCA)-biplot performed for g_s_ (1), E (2), VPD (3), PhiPS2 (4), ETR (5), 3D leaf area (6), digital biomass (7), plant heigh average (8), SPAD (9), GLI (10), NDVI(11), NPCI (12), PSRI (13), SR (14), EVI (15), SAVI (16), PRI (17), MASVI2 (18), WBI (19), NDWI (20), dry shoot biomass (21), dry root biomass (22), root index (23), A (24), WUE (25), C_i_ (26) values of *Ligustrum vulgare.* The labels in red capital letters represent the centroids of the sample clouds for each treatment represented by C, S1, S2, W2, S1W2, and S2W2.

Principal component analyses (PCAs) were performed to facilitate the visualization of treatment-related differences across a comprehensive set of morphophysiological, biometric, and spectral variables, including gas exchange parameters, chlorophyll fluorescence traits, structural descriptors, vegetation indices, and biomass-related metrics (**Figures 4**, **5**, **6**). The PCA ordinations consistently separated treatments across the three *Ligustrum* species (*L. sinense*, *L. japonicum* ‘Texanum’, and *L. vulgare*) based on their overall morphophysiological responses. In *L. sinense* (**Figure 4**) (PC1 explaining 36.68% and PC2 explaining 22.06% of the variation), the samples related to control treatment (C) were mainly plotted for positive values of PC1 and positively related to 3D leaf area (0.293), digital biomass (0.283), and root dry biomass (0.287). While W2 samples, mainly occupied the quadrants characterized by negative values of PC1 and related to NPCI (-0.241), GLI (-0.229), and ETR (-0.217). In *L. japonicum* ‘Texanum’ (**Figure 5**) (PC1 explaining 28.08% and PC2 explaining 15.20% of the variation), the control treatment (C) was likewise positioned in the first quadrant, but together with treatment W2. Treatment S1 was positioned in the second quadrant, S1W2 in the third, and S2 in the fourth quadrant. Based on the factor loadings, the variables that most strongly contributed to the separation between W2 and C along PC1 were SAVI (0.330), MSAVI2 (0.314), and WBI (0.286). In treatment S1, values of PC1 were positively related to 3D leaf area (0.300) and digital biomass (0.290), whereas PC2 was negatively associated with net CO_2_ assimilation rate, A (-0.370), and WUE (-0.398). In treatment S1W2, PC1 values were negatively associated with NDWI (-0.250), NPCI (-0.173), and PSRI (-0.162). At the end, treatment S2, values of PC2 were positively related to C_i_ (0.399), dry root biomass (0.261), and transpiration (0.309). In *L. vulgare* (**Figure 6**) (PC1 explaining 32.59% and PC2 explaining 12.58% of the variation), treatment C and W2 were located in the first quadrant, S2 and S1W2 in the third quadrant, and S1 and S2W2 in the fourth quadrant. The samples related to C and W2 were mainly plotted for positive values of PC1 and related to A (0.273), WUE (0.254), and SAVI (0.246). Treatment S1W2 and S2 were plotted for negative values of PC1 related to C_i_ (-0.252). Treatments S1 and S2W2 were plotted for a negative values of PC1 related to PSRI (-0.201) and NPCI (-0.209).

## 4 Discussion

The cultivation of native or non-native non-invasive ornamental species tolerant of numerous stress factors has become a priority in contemporary urban green design (Mircea et al., 2024). In this study, the invasive species *L. sinense*, the native species *L. vulgare*, and the non-native non-invasive ornamental cultivar *L. japonicum* ‘Texanum’ exhibited different morphophysiological responses to salinity, drought stress, and their combined effect. Our study adopts a comparative approach between *Ligustrum* species with different ecological statuses (native and invasive) in a simulated urban stress context. The results contribute to expanding the gap in data on morpho-physiological responses to drought and salt stress combinations. The use of new and advanced phenotyping platforms (PhenoPlant) improves the results in the literature, which are limited. In addition, the study provides useful results for selecting native and non-native non-invasive alternatives to invasive ornamental species in the European context. Abiotic stress factors are known to induce divergent responses not only among species within the same genus (Al Hassan et al., 2016) but also among different cultivars (Cicevan et al., 2016), often depending on the ecological and geographical origins of the species. Considering the phenotypic plasticity of invasive plants, they may be more resistant to abiotic stress conditions (Mircea et al., 2023a), but conversely, invasiveness does not automatically guarantee stress tolerance, and some invasive species only exhibit invasiveness under favorable conditions (Mircea et al., 2023b). Indeed, in our study, the invasive species *L. sinense* survived, unexpectedly, only moderate water stress, while its resistance to salinity proved to be absent, contrary to what was previously reported by Caser et al. (2011). It should be noted that the growing conditions were different: while Caser et al. (2011) conducted their experiment in a greenhouse, our study was carried out in a growth chamber.

The use of PheoPlant platform allowed for a more detailed assessment of morphological traits than conventional 2D methods (Behmann et al., 2016). Previous studies used it to quantify morpho-physiological and vegetation index variations between plants under drought and salt stress conditions (Lazarević et al., 2021; Tripodi et al., 2024). The discussion focuses mainly at time of 90 days to avoid redundancy and highlight the established responses of plants to treatments. In *L. sinense*, the apparent increase observed under W2 conditions, from 60 to 90 days, probably reflects stress-induced deformations. Since growth is a direct indicator of stress, high-precision 3D measurement tools are suitable for detecting such stress by measuring changes in 3D shape (Paulus, 2019). As shown by Paulus et al. (2014), drought stress modifies leaf geometry by detecting deformations or inclinations that could lead to a change in apparent leaf area. The increase in 3D leaf area led to an increase in digital biomass, as it is the product of the two parameters (Lazarević et al., 2021). Despite this increase, drought stress conditions limited morphological parameters, as expected and demonstrated in other ornamental species such as *Ligustrum* and *Lantana* (Toscano et al., 2018). In moderate drought stress, the reduction of morphological parameters was accompanied by a decrease in NDVI and SPAD value, indicating a decline in chlorophyll content. NDVI is widely used to assess vegetation health (Kior et al., 2021) and is correlated with chlorophyll content: an increase in NDVI is generally associated with higher chlorophyll content (Wang et al., 2023). However, drought stress did not always lead to a decrease in chlorophyll content; For example, Toscano et al. (2018) reported no differences in chlorophyll content in *Ligustrum* species under different water regimes. The physiological responses observed suggest that *L. sinense* under moderate drought stress adopted a much more conservative strategy characterized by stomatal closure and reduced transpiration. This response is commonly reported under drought stress in several ornamental species, including *Ligustrum lucidum*, *Lantana camara*, *Polygala myrtifolia*, and *Viburnum lucidum* (Toscano et al., 2018; Tribulato et al., 2019). Reduced stomatal conductance (g_s_) and a decrease in photosynthesis content led to a decrease in dry shoot biomass, as demonstrated in drought-stressed plants of *Ligustrum obtusfiolium* (Wang et al., 2024), and affected root biomass and their structure (Kang et al., 2022).

In *L. japonicum* ‘Texanum’, the general reduction in morphological parameters under stress treatments, compared with non-stressed plants, represents a common response (Toscano et al., 2021; Cocozza et al., 2025). Drought and salt stress exhibited similar effects on morphological parameters. This result was consistent with the results reported by Angon et al. (2022), who observed comparable growth responses under water and salt stress conditions. This may reflect an early response phase, in which both drought and salt stress mainly induced drought stress, while prolonged exposure leads to ionic stress, resulting in more pronounced differences between the two stress conditions (Ma et al., 2020). In the S1W2 combination, a more pronounced response was expected than for individual stresses (Angon et al., 2022), as was also the case in *Callistemon* and *Viburnum* plants (Toscano et al., 2021). In our study, the insignificant difference suggested that moderate drought could have saturated the stress response mechanisms (Zandalinas and Mittler, 2022), given that those in response to salt stress are not immediate (Ma et al., 2020). The increase in digital biomass under S2, from 60 to 90 days, despite reduced leaf area, may reflect vertical growth contributing disproportionately to 3D measurements (Lazarević et al., 2021). Reflectance indices such as NPCI express the ratio between non-photosynthetic pigments and chlorophyll and are therefore sensitive to senescence and stress, typically increasing because of chlorophyll degradation (Kior et al., 2021; Song and Wang, 2022). The stresses applied indicated general pigment stability, but the S1W2 combination showed greater pigment alteration than the single salt stress, consistent with a relative increase in carotenoids and/or a reduction in chlorophyll under combined stress (Dugasa et al., 2019). High salinity (S2) had a greater impact on water, as indicated by the WBI, than moderate water stress, as it reduces the ability of plants to absorb water. Moreover, the combination of salinity with drought (S1W2) caused a sharp decline in WBI values, suggesting a reduced water uptake capacity at the root level and, consequently, the induction of stomatal closure (Peñuelas et al., 1997a; Fu and Yang, 2023). High NDWI values in S2 and S1W2 values may reflect not only the water content of the canopy but also the contributions of water not strictly bound to plant tissues and may be influenced by soil water status and canopy structure (Zhou et al., 2022). Differences between stomatal conductance measurements obtained with IRGA and a porometer indicate that g_s_ may vary not only with environmental conditions but also as a function of the instrument used (Toro et al., 2019). Moderate drought stress maintained relatively high chlorophyll content, as reported in previous studies (Toscano et al., 2018); however, its combination with salinity (S1) resulted in a significant decrease in chlorophyll content (Angon et al., 2022). The high values of g_s_ and transpiration observed under moderate drought stress indicate a response that contrasts with that reported by Zuccarini et al. (2020), in which *Ligustrum japonicum* plants showed marked sensitivity to drought stress, with a reduction in physiological performance. Saline treatments maintained stable chlorophyll values, typical of salt-tolerant species (Liu et al., 2024). Furthermore, the S2 treatment recorded the highest g_s_ and transpiration values. Although there is currently no specific literature indicating that these responses represent a compensatory mechanism under salt stress, it is reported that more salt-tolerant plants tend to maintain higher g_s_ values under saline conditions compared to sensitive species (Boussora et al., 2024; Nguyen and Stangoulis, 2024). In general, salt stress is known to induce Na^+^ mediated stomatal closure, leading to photosynthetic imbalance and reduced photosynthetic performance (Boussora et al., 2024). *L. japonicum* ‘Texanum’ adopted a strategy of preferential biomass allocation to the roots, as no significant differences in root biomass and root structural architecture were observed compared to the control. Similar results were also reported by Silva et al. (2012), who demonstrated that *Ligustrum japonicum* plants subjected to drought stress reduced shoot development, while root dry mass remained unchanged. The maintenance of root structure and biomass under water stress reflects the ability of roots to adapt in order to preserve water uptake (Kim et al., 2020). This can also occur under salinity conditions, favoring the allocation of resources to the root system as an adaptive strategy to safeguard water functions in the early stages of stress (Albacete et al., 2008; Karlova et al., 2021), such as an increase in endogenous ABA (Teng et al., 2023).

In *L. vulgare*, salinity was the main limiting factor for 3D leaf area and digital biomass, proving more significant than moderate drought stress, especially in combinations with salt × drought (Angon et al., 2022). Three-dimensional phenotyping studies on basil have shown a marked reduction in leaf area under salinity conditions (Lazarević et al., 2021). Similarly, Toscano et al. (2020) reported significant decreases in morphological parameters in several ornamental shrubs exposed to salt stress. In *L. vulgare*, the maintenance of high values of morphological traits, such as 3D leaf area and digital biomass, under moderate drought stress conditions (W2), despite a reduction in dry biomass, suggests the adoption of a conservative structural strategy. This response contrasts with the results of Cocozza et al. (2025) in pepper plants, where 3D phenotyping revealed a decrease in morphological parameters under drought stress, and also differed from the results reported by Toscano et al. (2018), who observed that moderate drought stress did not affect dry biomass in *Ligustrum lucidum*. In *L. vulgare*, vegetation indices showed a clear differentiation between treatments in terms of canopy vigor, pigment content, and senescence dynamics. The single saline treatments (S1, S2) and the S2W2 combination showed a marked reduction in canopy vigor (lower NDVI), consistent with a decrease in chlorophyll content (Wang et al., 2023). Salt stress compromised chlorophyll content (Shin et al., 2021), and in the S2W2 combination, the high NPCI value compared to W2 and S1W2 reflects the marked reduction in chlorophyll content (Kior et al., 2021; Song and Wang, 2022; Patil and Mali, 2025). S2 and S2W2 had higher PSRI values than W2, indicating an imbalance in the carotenoid-to-chlorophyll ratio and an indicator of leaf senescence (Kior et al., 2021; Song & Wang, 2022), while under moderate drought stress conditions, NDVI was negatively correlated with NPCI and PSRI (Leporino et al., 2024), indicating good pigment status. Overall, this indicates that salt stress, more than moderate water stress, had more clearly compromised pigments and accelerated senescence processes and has also reducing the water status of plants (Peñuelas et al., 1997a; Gonzáles et al., 2021). Based on WBI values, moderate water stress did not compromise the water status of plants, suggesting a possible tolerance mechanism. WBI is a sensitive indicator for the early diagnosis of water stress (Penuelas et al., 1997b), unlike NDWI, which did not discriminate between treatments. Furthermore, WBI was significantly correlated with NDVI, indicating that higher vegetation water content was related to a higher NDVI value. NDVI values tend to increase with increasing water content in vegetation because water more easily reflects longer wavelength radiation (Yu et al., 2021). High water content may also reflect a greener canopy (Claudio et al., 2006). Physiological parameters showed differences in g_s_ and transpiration values between IRGA and porometer measurements (Toro et al., 2019). In general, saline treatments and combinations with moderate drought stress caused a sharp reduction in chlorophyll content compared to untreated plants, as expected (Angon et al., 2022; Umar et al., 2019). Osmotic stress causes oxidative damage with chlorophyll loss (Lu et al., 2023). In salt tretaments an increase in C_i_ is unexpected because with closed or semi-closed stomata, the amount of C_i_ would also decrease, but instead it remained high. This behavior, associated with reduced SPAD values, indicates a biochemical limitation of photosynthesis (Liu et al., 2024), as also observed in Prunus salicina (Ziska et al., 1990) and *Miscanthus* × *giganteus* (Stavridou et al., 2020). Under moderate drought stress conditions, *L. vulgare* maintained good assimilation rates and efficient photosynthetic activity, as reported by Toscano et al. (2018) in other *Ligustrum* spp. species and by Tattini et al. (2004), who demonstrated that *L. vulgare* activated effective physiological responses under drought conditions, indicating good adaptability. However, since the results were obtained under controlled conditions, stress tolerance seems to depend largely on the environmental context (Guidi et al., 2008). Salt stress and its combination with moderate drought stress compromised root development and overall growth (Angon et al., 2022; Fu and Yang, 2023). Moderate drought stress maintained good root architecture similar to untreated plants, which can be considered an adaptive trait to drought in many species: total mass is reduced, but functional traits that support water absorption are maintained or optimized (Kou et al., 2022; Kang et al., 2022).

In conclusion, moderate drought stress was generally tolerated better than salt stress and combined conditions, highlighting different adaptation strategies among species. Although the results confirm the use of *L. japonicum* ‘Texanum’ and *L. vulgare* as alternatives to *L. sinense* in urban environments, the research showed some limitations that create opportunities for future research developments. A possible limitation of this study is the controlled environment used for the experiment, as well as the young age of the plants analyzed, which may have influenced the observed results. Future studies should therefore consider experiments in the field in real urban conditions, or in greenhouses, and on plants at more advanced stages of development, with the possibility of extending monitoring for several years. Nevertheless, the results highlight the potential of *L. japonicum* ‘Texanum’ and *L. vulgare* for urban greening, reinforcing the importance of promoting non-invasive ornamental or native species that are resilient to the environmental challenges typical of urban contexts.

## Data Availability

The raw data supporting the conclusions of this article will be made available by the authors, without undue reservation.

## Footnotes

1 https://www.eea.europa.eu/signals-archived/signals-2018-content-list/articles/water-use-in-europe-2014. (Accessed on October 8, 2025

2 https://www.mase.gov.it/pagina/riutilizzo-acque-reflue-relazione. (Accessed on October 8, 2025)

3 https://www.rhs.org.uk/plants/search-results?form-mode=true&query=ligustrum, (Accessed on 2 July 2025)

4 https://www.rhs.org.uk/plants/45594/ligustrum-japonicum-texanum/details. (Accessed on 2 July 2025)

5 https://powo.science.kew.org/taxon/urn:lsid:ipni.org:names:610130-1#distributions. (Accessed on 5 November 2025).

6 https://www.rhs.org.uk/plants/10108/ligustrum-vulgare/details. (Accessed on 2 July 2025)

7 https://www.rhs.org.uk/plants/81603/ligustrum-sinense/details. (Accessed on 2 July 2025)

8 https://www.rhs.org.uk/plants/10108/ligustrum-vulgare/details. (Accessed on 2 July 2025)

9 https://www.rhs.org.uk/plants/45594/ligustrum-japonicum-texanum/details. (Accessed on 2 July 2025)

10 https://www.rhs.org.uk/gardens/wisley (Accessed on 2 July 2025)

11 Plant phenotyping platform - Dipartimento di Scienze Agrarie, Forestali e Alimentari - DISAFA - Università degli Studi di Torino (accessed on 28 January, 2026)
