# Supplementary material for: Evaluation of morpho-physiological responses to drought and salt stress in two ornamental alternatives to Invasive Ligustrum sinense Lour
Source: Front Plant Sci. 2026 Apr 24;17:1770722. doi: 10.3389/fpls.2026.1770722 (PMC13152734; doi:10.3389/fpls.2026.1770722)
Supplement: Supplementary file 1 [file Table1.docx]

Supplementary Material

# Supplementary Tables

**Table S1**. The mortality rates at 30 (T30), 60 (T60), and 90 days (T90) were assessed for *Ligustrum sinense*, *Ligustrum japonicum* ‘Texanum’, and *Ligustrum vulgare*. The treatments applied included: control (C), salt stress at 150 mM NaCl (S1), salt stress at 300 mM NaCl (S2), water stress at 30% pot capacity (W1), water stress at 60% pot capacity (W2), a combination of 150 mM NaCl and 30% pot capacity (S1W1), a combination of 150 mM NaCl and 60% pot capacity (S1W2), a combination of 300 mM NaCl and 30% pot capacity (S2W1), and a combination of 300 mM NaCl and 60% pot capacity (S2W2). Different letters indicate significant differences among species and treatments (Tukey’s test, *p* < 0.05). Significance levels: ****p* < 0.001 (ANOVA). Values within the same column followed by the same letter are not significantly different.

|  | *Ligustrum. sinense* | | | | *Ligustrum japonicum.* ‘Texanum’ | | | *Ligustrum vulgare* | | |
| --- | --- | --- | --- | --- | --- | --- | --- | --- | --- | --- |
| **Treatments** | | **T30 %** | **T60 %** | **T90 %** | **T30 %** | **T60 %** | **T90 %** | **T30 %** | **T60 %** | **T90 %** |
| C | | 0 ^d^ | 0 ^c^ | 0 ^c^ | 0 ^b^ | 0 ^c^ | 0 ^c^ | 0 ^b^ | 0 ^b^ | 0 ^b^ |
| S1 | | 40 ^c, d^ | 90 ^a^ | 90 ^a^ | 10 ^b^ | 10 ^b, c^ | 20 ^b, c^ | 10 ^b^ | 10 ^b^ | 10 ^b^ |
| S2 | | 90 ^a, b^ | 100 ^a^ | 100 ^a^ | 30 ^b^ | 40 ^b^ | 50 ^b^ | 0 ^b^ | 0 ^b^ | 20 ^b^ |
| W1 | | 90 ^a, b^ | 100 ^a,^ | 100 ^a^ | 100 ^a^ | 100^,^ ^a^ | 100^,^ ^a^ | 80 ^a^ | 100 ^a^ | 100 ^a^ |
| W2 | | 30 ^c, d^ | 30 ^b^ | 30 ^b^ | 0 ^b^ | 0 ^c^ | 0 ^c^ | 0 ^b^ | 0 ^b^ | 10 ^b^ |
| S1W1 | | 90 ^a, b^ | 100 ^a^ | 100 ^a^ | 90 ^a^ | 100 ^a^ | 100 ^a^ | 10 ^b^ | 100 ^a^ | 100 ^a^ |
| S1W2 | | 50 ^b, c^ | 80 ^a^ | 100 ^a^ | 10 ^b^ | 10 ^c^ | 20 ^b, c^ | 0 ^b^ | 0 ^b^ | 20 ^b^ |
| S2W1 | | 100 ^a^ | 100 ^a^ | 100 ^a^ | 70 ^a^ | 100 ^a^ | 100 ^a^ | 0 ^b^ | 100 ^a^ | 100 ^a^ |
| S2W2 | | 100 ^a^ | 100 ^a^ | 100 ^a^ | 80 ^a^ | 80 ^a^ | 100 ^a^ | 20 ^b^ | 20 ^b^ | 40 ^b^ |
| *Sig.* | | ***** | ***** | ***** | ***** | ***** | ***** | ***** | ***** | ***** |

**Table S2.** At the midpoint of the experiment, morphological parameters were assessed for *Ligustrum sinense*, *Ligustrum japonicum* ‘Texanum’, and *Ligustrum vulgare*. These parameters included three-dimensional leaf area (mm²), digital biomass (mm³) and plantheight average (mm). The treatments applied included: control group (C), salt stress conditions at 150 mM NaCl (S1) and 300 mM NaCl (S2), water stress at 60% pot capacity (W2), a combination of 150 mM NaCl and 60% pot capacity (S1W2), and a combination of 300 mM NaCl and 60% pot capacity (S2W2). Different letters indicate significant differences among species and treatments (Tukey’s test, *p* < 0.05). Significance levels: **p* < 0.05; ***p* < 0.01; ***p* < 0.001 (ANOVA). Values within the same column followed by the same letter are not significantly different.

| **Species** | **3D Leaf Area (mm^2^)** | **Digital Biomass**  **(mm^3^)** | **Plant Height Average(mm)** | | |
| --- | --- | --- | --- | --- | --- |
| *Ligustrum sinense* | 14870^a^ | 3270029 ^a,b^ | | 210 ^b^ |  |
| *Ligustrum japonicum* ‘Texanum’ | 13431 ^a^ | 2416231 ^b^ | 181 ^b^ | | |
| *Ligustrum vulgare* | 11094 ^b^ | 3972143 ^a^ | 332 ^a^ | | |
| *Sig*. | *** | *** | *** | | |
| **Treatments** |  |  |  | | |
| C | 19875 ^a^ | 5254712 ^a^ | 271 | | |
| S1 | 11217.9 ^b^ | 2615114 ^b^ | | 250 |  |
| S2 | 11006 ^b^ | 3102662 ^b^ | 276 | | |
| W2 | 10119 ^b^ | 2846478 ^b^ | 252 | | |
| S1W2 | 8481 ^b^ | 2105346 ^b^ | 235 | | |
| S2W2 | 7495 ^b^ | 1865828 ^b^ | 217 | | |
| *Sig.* | *** | *** | *** | | |
| *Species*Treatments* | ***** | ***** | *** | | |

**Table S3.** The morphological parameters assessed at the conclusion of the experiment for *Ligustrum sinense*, *Ligustrum japonicum* ‘Texanum’, and *Ligustrum vulgare* included three-dimensional leaf area (mm²), digital biomass (mm³), and average plant height (mm). The treatments applied included: control (C), salt stress at 150 mM NaCl (S1), salt stress at 300 mM NaCl (S2), water stress at 60% pot capacity (W2), a combination of 150 mM NaCl and 60% pot capacity (S1W2), and a combination of 300 mM NaCl and 60% pot capacity (S2W2). Different letters indicate significant differences among species and treatments (Tukey’s test, *p* < 0.05). ns = not significant. Significance levels: **p* < 0.05; ****p* < 0.001 (ANOVA). Values within the same column followed by the same letter are not significantly different.

| **Species** | **3D Leaf Area (mm^2^)** | **Digital Biomass (mm^3^)** | **Plant Height Averaged (mm)** |
| --- | --- | --- | --- |
| *Ligustrum sinense* | 24977 ^a^ | 5700499 ^a^ | 213 ^b^ |
| *Ligustrum japonicum* ‘Texanum’ | 14024 ^b^ | 2681452 ^b^ | 187 ^c^ |
| *Ligustrum vulgare* | 8916 ^c^ | 3352906 ^b^ | 350 ^a^ |
| *Sig.* | ***** | *** | ***** |
| Treatments |  |  |  |
| C | 26277 ^a^ | 6551650 ^a^ | 276 ^a,b^ |
| S1 | 8648 ^b,c,d^ | 2003050 ^b,c^ | 257 ^a,b^ |
| S2 | 9007 ^b,c^ | 3301705 ^b^ | 336 ^a^ |
| W2 | 11017 ^b^ | 2885296 ^b,c^ | 249 ^a,b^ |
| S1W2 | 5389 ^c,d^ | 1374159 ^b,c^ | 241 ^b^ |
| S2W2 | 2706 ^d^ | 948587 ^c^ | 257 ^a,b^ |
| *Sig.* | ***** | ***** | **** |
| *Species*Treatments* | ***** | ***** | ns |

**Table S5.** The vegetation indices assessed at the midpoint of the experiment for *Ligustrum sinense*, *Ligustrum japonicum* ‘Texanum’, and *Ligustrum vulgare* included the green leaf index (GLI), normalized difference vegetation index (NDVI), normalized pigment chlorophyll ratio index (NPCI), and plant senescence reflectance index (PSRI). The treatments applied included: control (C), salt stress at 150 mM NaCl (S1), salt stress at 300 mM NaCl (S2), water stress at 60% pot capacity (W2), a combined treatment of 150 mM NaCl and 60% pot capacity (S1W2), and a combined treatment of 300 mM NaCl and 60% pot capacity (S2W2). Different letters indicate statistically significant differences among species and treatments (Tukey’s test, *p* < 0.05). ns = not significant. Significance levels: **p* < 0.05; ***p* < 0.01; ****p* < 0.001 (ANOVA). Values within the same column followed by the same letter are not significantly different.

| **Species** | **GLI** | **NDVI** | **NPCI** | **PSRI** |
| --- | --- | --- | --- | --- |
| *Ligustrum sinense* | 0.178 ^c^ | 0.587 ^b^ | 0.000 ^c^ | 0.050^a^ |
| *Ligustrum japonicum* ‘Texanum’ | 0.340 ^a^ | 0.651 ^a^ | 0.181 ^a^ | 0.098^b^ |
| *Ligustrum vulgare* | 0.261 ^b^ | 0.564^b^ | 0.144 ^b^ | 0.143^c^ |
| *Sig.* | *** | *** | *** | * |
| **Treatments** |  |  |  |  |
| C | 0.270 ^b,c^ | 0.673 ^a^ | 0.037 ^b^ | 0.041 ^c^ |
| S1 | 0.309 ^a,b^ | 0.598 ^a,b^ | 0.191 ^a^ | 0.109 ^b,c^ |
| S2 | 0.325 ^a^ | 0.560 ^b^ | 0.235 ^a^ | 0.140 ^b^ |
| W2 | 0.231 ^c^ | 0.598 ^a,b^ | 0.081 ^b^ | 0.092 ^b,c^ |
| S1W2 | 0.265 ^b,c^ | 0.528 ^b^ | 0.196 ^a^ | 0.218 ^a^ |
| S2W2 | 0.325 ^a^ | 0.565 ^b^ | 0.227 ^a^ | 0.156 ^a,b^ |
| *Sig.* | ** | *** | *** | *** |
| *Species*Treatments* | ns | ns | ns | ns |

**Table S6.** The vegetation indices assessed at the end of the experiment for *Ligustrum sinense*, *Ligustrum japonicum* ‘Texanum’, and *Ligustrum vulgare* included the green leaf index (GLI), normalized difference vegetation index (NDVI), normalized pigment chlorophyll ratio index (NPCI), and plant senescence reflectance index (PSRI). The treatments applied included: control (C), salt stress at 150 mM NaCl (S1), salt stress at 300 mM NaCl (S2), water stress at 60% pot capacity (W2), a combined treatment of 150 mM NaCl and 60% pot capacity (S1W2), and a combined treatment of 300 mM NaCl and 60% pot capacity (S2W2). Different letters indicate significant differences among species and treatments (Tukey’s test, *p* < 0.05). ns = not significant. Significance levels: **p* < 0.05; ***p* < 0.01; ****p* < 0.001 (ANOVA). Values within the same column followed by the same letter are not significantly different.

| **Species** | **GLI** | **NDVI** | **NPCI** | **PSRI** |
| --- | --- | --- | --- | --- |
| *Ligustrum sinense* | 0.182 ^c^ | 0.663 ^a^ | -0.054 ^c^ | 0.019 ^b^ |
| *igustrum japonicum* ‘Texanum’ | 0.301 ^a^ | 0.680 ^a^ | 0.084 ^b^ | 0.042 ^b^ |
| *Ligustrum vulgare* | 0.247 ^b^ | 0.555 ^b^ | 0.139 ^a^ | 0.095 ^a^ |
| *Sig.* | *** | *** | * | ** |
| Treatments |  |  |  |  |
| C | 0.246 | 0.662 ^a,b^ | 0.030 ^c^ | 0.040 ^b,c^ |
| S1 | 0.252 | 0.591 ^b,c^ | 0.097 ^b,c^ | 0.083 ^b^ |
| S2 | 0.270 | 0.560 ^c^ | 0.151 ^b^ | 0.098 ^b^ |
| W2 | 0.246 | 0.673 ^a^ | 0.022 ^c^ | 0.021 ^c^ |
| S1W2 | 0.299 | 0.594 ^b,c^ | 0.157 ^b^ | 0.077 ^b^ |
| S2W2 | 0.229 | 0.463 ^d^ | 0.273 ^a^ | 0.170 ^a^ |
| *Sig.* | Ns | *** | *** | *** |
| *Species*Treatments* | ns | *** | ***** | **** |

**Table S7*.*** The vegetation indices assessed at the midpoint of the experiment for *Ligustrum sinense*, *Ligustrum japonicum* ‘Texanum’, and *Ligustrum vulgare* included the simple ratio (SR), enhanced vegetation index (EVI), soil-adjusted vegetation index (SAVI), modified soil-adjusted vegetation index (MSAVI2), photochemical reflectance index (PRI), water band index (WBI), and normalized difference water index (NDWI). The treatments applied included: control (C), salt stress at 150 mM NaCl (S1), salt stress at 300 mM NaCl (S2),water stress at 60% pot capacity (W2), combined treatment of 150 mM NaCl and 60% pot capacity (S1W2), and combined treatment of 300 mM NaCl and 60% pot capacity (S2W2). Different letters indicate significant differences among species and treatments (Tukey’s test, *p* < 0.05). ns = not significant. Significance levels: **p* < 0.05; ***p* < 0.01; ****p* < 0.001 (ANOVA). Values within the same column followed by the same letter are not significantly different.

| **Species** | **SR** | **EVI** | **SAVI** | **PRI** | **MSAVI2** | **WBI** | **NDWI** |
| --- | --- | --- | --- | --- | --- | --- | --- |
| *Ligustrum sinense* | 15.539 ^a^ | 0.933 ^a,b^ | 0.699 ^b^ | 0.060 ^a^ | 1.242 ^b^ | 0.991 ^a^ | 0.034 ^c^ |
| *Ligustrum japonicum* ‘Texanum’ | 11.975 ^b^ | 1.003 ^a^ | 0.775 ^a^ | 0.023 ^b^ | 1.298 ^a^ | 0.947 ^c^ | 0.095 ^a^ |
| *Ligustrum vulgare* | 7.544 ^c^ | 0.886 ^b^ | 0.673 ^b^ | 0.013 ^b^ | 1.192 ^b^ | 0.976 ^b^ | 0.057 ^b^ |
| Sig. | *** | ** | *** | ** | *** | *** | *** |
| Treatments |  |  |  |  |  |  |  |
| C | 13.324 ^a^ | 1.006 ^a^ | 0.748 | 0.045 ^a^ | 1.281 | 0.981 ^a^ | 0.054 ^c^ |
| S1 | 8.714^b^ | 0.982 ^a,b^ | 0.722 | 0.022 ^a,b^ | 1.236 | 0.961 ^c^ | 0.074 ^b^ |
| S2 | 7.552 ^b^ | 0.826 ^b^ | 0.676 | -0.002 ^b^ | 1.192 | 0.953 ^d^ | 0.090 ^a^ |
| W2 | 13.581 ^a^ | 0.951 ^a,b^ | 0.735 | 0.043 ^a^ | 1.271 | 0.976 ^a,b^ | 0.056 ^c^ |
| S1W2 | 8.045^b^ | 0.918 ^a,b^ | 0.696 | 0.012^b^ | 1.212 | 0.951 ^d^ | 0.083 ^a,b^ |
| S2W2 | 6.887 ^b^ | 0.875 ^a,b^ | 0.675 | 0.001^b^ | 1.192 | 0.972 ^b^ | 0.057 ^c^ |
| *Sig.* | * | * | ns | *** | ns | *** | *** |
| *Species*Treatments* | ns | ns | ns | ns | ns | ***** | ***** |

**Table S8.** The vegetation indices assessed at the end of the experiment for *Ligustrum sinense*, *L. japonicum* ‘Texanum’, and *L. vulgare* included the simple ratio (SR), enhanced vegetation index (EVI), soil-adjusted vegetation index (SAVI), modified soil-adjusted vegetation index (MSAVI2), photochemical reflectance index (PRI), water band index (WBI), and normalized difference water index (NDWI). The treatments applied were as follows: control (C), salt stress at 150 mM NaCl (S1), salt stress at 300 mM NaCl (S2), water stress at 60% pot capacity (W2), combined treatment of 150 mM NaCl and 60% pot capacity (S1W2), and combined treatment of 300 mM NaCl and 60% pot capacity (S2W2). Different letters indicate significant differences among species and treatments (Tukey’s test, *p* < 0.05). ns = not significant. Significance levels: **p* < 0.05; ***p* < 0.01; ****p* < 0.001 (ANOVA). Values within the same column followed by the same letter are not significantly different.

| **Species** | **SR** | **EVI** | **SAVI** | **PRI** | **MSAVI2** | **WBI** | **NDWI** |
| --- | --- | --- | --- | --- | --- | --- | --- |
| *Ligustrum sinense* | 15.861 ^a^ | 1.013 ^a^ | 0.748 ^b^ | 0.071 ^a^ | 1.290 ^a^ | 0.985 ^a^ | 0.041 ^c^ |
| *Ligustrum japonicum* ‘Texanum’ | 13.651 ^a^ | 1.037 ^a^ | 0.802 ^a^ | 0.034 ^b^ | 1.329 ^a^ | 0.942 ^c^ | 0.096 ^a^ |
| *Ligustrum vulgare* | 7.997 ^b^ | 0.893 ^b^ | 0.681 ^c^ | 0.015 ^c^ | 1.200 ^b^ | 0.973 ^b^ | 0.054 ^b^ |
| Sig. | *** | ** | *** | *** | *** | *** | *** |
| Treatments |  |  |  |  |  |  |  |
| C | 14.368 ^a^ | 1.067 ^a^ | 0.787 ^a^ | 0.055 ^a^ | 1.321 ^a^ | 0.972 ^a^ | 0.058 ^b,c^ |
| S1 | 11.519 ^a,b,c^ | 0.925 ^a,b^ | 0.708 ^b^ | 0.031 ^a,b^ | 1.229 ^b^ | 0.959 ^b^ | 0.072 ^a,b^ |
| S2 | 7.554 ^c,d^ | 0.859 ^b^ | 0.677 ^b^ | 0.005 ^b^ | 1.192 ^b^ | 0.960 ^b^ | 0.079 ^a^ |
| W2 | 13.355 ^a,b^ | 1.067 ^a^ | 0.785 ^a^ | 0.053 ^a^ | 1.317 ^a^ | 0.972 ^a^ | 0.057 ^b,c^ |
| S1W2 | 9.308 ^b,c,d^ | 0.856 ^b^ | 0.698 ^b^ | 0.014 ^b^ | 1.221 ^b^ | 0.950 ^b^ | 0.082 ^a^ |
| S2W2 | 7.147 ^d^ | 0.840 ^b^ | 0.670 ^b^ | -0.022 ^c^ | 1.190 ^b^ | 0.970 ^a^ | 0.055 ^c^ |
| *Sig.* | * | *** | *** | *** | *** | *** | ** |
| *Species*Treatments* | ns | ns | ns | ns | ns | ***** | *** |

**Table S9.**  Dry shoot biomass (g), root dry biomass (g), and root index were evaluated at the conclusion of the experiment for *Ligustrum sinense*, *L. japonicum* ‘Texanum’, and *L. vulgare*. The Root Index was measured on a scale from 0 to 3, where 0 represents 0–25%, 1 represents 26–50%, 2 represents 51–75%, and 3 represents 76–100% of pot volume colonized by roots. The treatments survived were as follows: control (C), salt stress at 150 mM NaCl (S1), salt stress at 300 mM NaCl (S2), water stress at 60% pot capacity (W2), 150 mM NaCl and 60% pot capacity (S1W2), and 300 mM NaCl and 60% pot capacity (S2W2). Different letters indicate statistically significant differences among species and treatments (Tukey’s test, *p* < 0.05). **p* < 0.05; ***p* < 0.01; ****p* < 0.001 denote significance levels of main effects according to ANOVA. Values within the same column followed by the same letter are not significantly different.

| **Species** | **Dry shoot biomass (g)** | **Roots dry**  **weight (g)** | **Root index** |
| --- | --- | --- | --- |
| *Ligustrum sinense* | 5.4 ^a^ | 1.7 ^c^ | 2.6 ^a^ |
| *Ligustrum japonicum* ‘Texanum’ | 5.1^a,b^ | 2.8 ^a^ | 2.7 ^a^ |
| *Ligustrum vulgare* | 4.5 ^b^ | 2.3 ^b^ | 1.9 ^b^ |
| Sig. | *** | *** | *** |
| Treatment |  |  |  |
| C | 8.7 ^a^ | 3.0 ^a^ | 2.9 ^a^ |
| S1 | 4.0^b,c^ | 2.4 ^a,b^ | 2.3 ^b,c^ |
| S2 | 3.4 ^b,c^ | 2.7 ^a,b^ | 2.1 ^b,c^ |
|  |  |  |  |
| W2 | 4.5 ^b^ | 2.1 ^b,c^ | 2.6 ^a,b^ |
|  |  |  |  |
| S1W2 | 2.9 ^c,d^ | 2.25 ^a,b,c^ | 1.8 ^c^ |
|  |  |  |  |
| S2W2 | 2.1 ^d^ | 1.6 ^c^ | 1.1 ^c^ |
| *Sig.* | *** | *** | *** |
| Species * Treatments | * | ** | *** |

**Table S10*.*** 30 days after the initiation of the experiment, physiological parameters were assessed for *Ligustrum sinense*, *L. japonicum* ‘Texanum’, and *L.* *vulgare*. These parameters included SPAD units, stomatal conductance (gₛ, mol m⁻² s⁻¹), transpiration rate (E mmol m⁻² s⁻¹), leaf vapor pressure deficit (VPD Leaf, kPa), photosystem II efficiency (PhiPS2), and electron transport rate (ETR, µmol electrons m⁻² s⁻¹). The treatments applied were as follows: control (C), salt stress at 150 mM NaCl (S1), salt stress at 300 mM NaCl (S2), water stress at 30% pot capacity (W1), water stress at 60% pot capacity (W2), and combined treatments of 150 mM NaCl with 30% pot capacity (S1W1), 150 mM NaCl with 60% pot capacity (S1W2), 300 mM NaCl with 30% pot capacity (S2W1), and 300 mM NaCl with 60% pot capacity (S2W2). Different letters indicate statistically significant differences among species and treatments (Tukey’s test, *p* < 0.05). ns = not significant; **p* < 0.05; ****p* < 0.001 denote significance levels of main effects according to ANOVA. Values within the same column followed by the same letter are not significantly different.

| **Species** | **SPAD** | **g_s_**  **mol m⁻² s⁻¹** | **E**  **mmol m⁻² s⁻¹** | **VPDLeaf**  **kPa** | **PhiPS2** | **ETR**  **µmol e⁻ m⁻² s⁻¹** |
| --- | --- | --- | --- | --- | --- | --- |
| *Ligustrum sinense* | 50.82 ^a^ | 0.010 ^b^ | 0.23 ^b^ | 2.46 ^a^ | 0.59 ^a,b^ | 54.56 |
| *Ligustrum japonicum* ‘Texanum’ | 47.85 ^a^ | 0.029^a^ | 0.59 ^a^ | 2.15 ^b^ | 0.53 ^b^ | 39.31 |
| *Ligustrum vulgare* | 35.19 ^c^ | 0.020 ^a,b^ | 0.42 ^a,b^ | 2.26 ^b^ | 0.62 ^a^ | 48.56 |
| Sig. | *** | ns | ns | *** | * | Ns |
| Treatments |  |  |  |  |  |  |
| C | 47.40 ^a^ | 0.04 ^a,b^ | 0.74 ^a,b^ | 2.28 ^a, b^ | 0.69 ^a^ | 46.26 ^a, b^ |
| S1 | 48.53 ^a^ | 0.01 ^b,c^ | 0.29 ^b,c^ | 2.44 ^a^ | 0.53 ^b^ | 55.15 ^a^ |
| S2 | 47.33 ^a^ | 0.04 ^a^ | 0.84 ^a^ | 2.16 ^b^ | 0.57 ^a, b^ | 9.47 ^b^ |
|  |  |  |  |  |  |  |
| W2 | 47.20 ^a^ | 0.01 ^c^ | 0.29 ^b,c^ | 2.37 ^a, b^ | 0.70 ^a^ | 51.41 ^a^ |
| S1W1 | 34.74 ^b,c^ | 0.01 ^c^ | 0.21 ^c^ | 2.14 ^b^ | 0.58 ^a, b^ | 70.05 ^a^ |
| S1W2 | 39.18 ^b^ | 0.02 ^a,b,c^ | 0.39 ^b,c^ | 2.33 ^a, b^ | 0.57 ^a,b^ | 66.38 ^a^ |
| S2W1 | 33.01 ^b,c^ | 0.01 ^b,c^ | 0.32 ^b,c^ | 2.24 ^a, b^ | 0.45 ^b^ | 40.29 ^a^ |
| S2W2 | 27.10 ^c^ | 0.01^c^ | 0.15^c^ | 1.88 ^c^ | 0.48 ^b^ | 46.59 ^a^ |
| *Sig.* | * | *** | *** | *** | *** | *** |
| Species*Treatments | * | *** | *** | ns | ns | *** |

**Table S11*.*** 60 days after the initiation of the experiment, physiological parameters were assessed for *Ligustrum sinense*, L. japonicum ‘Texanum’, and L. vulgare. These parameters included SPAD units, stomatal conductance (gₛ, mol m⁻² s⁻¹), transpiration rate (E, mmol m⁻² s⁻¹), leaf vapor pressure deficit (VPD Leaf, kPa), photosystem II efficiency (PhiPS2), and electron transport rate (ETR, µmol electrons m⁻² s⁻¹). The treatments applied were as follows: control (C), salt stress at 150 mM NaCl (S1), salt stress at 300 mM NaCl (S2), water stress at 60% pot capacity (W2), a combination of 150 mM NaCl and 60% pot capacity (S1W2), and a combination of 300 mM NaCl and 60% pot capacity (S2W2). Different letters indicate statistically significant differences among species and treatments (Tukey’s test, *p* < 0.05). ns = not significant; **p* < 0.05; ***p* < 0.01; ****p* < 0.001 denote significance levels of main effects according to ANOVA. Values within the same column followed by the same letter are not significantly different.

| **Species** | **SPAD** | **g_s_**  **mol m⁻² s⁻¹** | **E**  **mmol m⁻² s⁻¹** | **VPDLeaf**  **kPa** | **PhiPS2** | **ETR**  **µmol e⁻ m⁻² s⁻¹** |
| --- | --- | --- | --- | --- | --- | --- |
| *Ligustrum sinense* | 62.36 ^a^ | 0.028 | 0.73 | 2.58 ^a^ | 0.65 | 70.79 |
| *Ligustrum japonicum* ‘Texanum’ | 52.16 ^b^ | 0.046 | 1.04 | 2.26 ^b^ | 0.53 | 58.38 |
| *Ligustrum vulgare* | 41.61 ^c^ | 0.047 | 1.04 | 2.31 ^a,b^ | 0.51 | 60.22 |
| *Sig*. | *** | ns | ns | * | ns | ns |
| Treatments |  |  |  |  |  |  |
| C | 62.05 ^a^ | 0.026 ^b^ | 0.59 ^b^ | 2.66 ^a^ | 0.59 ^a^ | 68.16 ^a^ |
| S1 | 45.06 ^b^ | 0.085 ^a^ | 1.80 ^a^ | 2.10 ^b^ | 0.56 ^a,b^ | 57.12 ^a,b^ |
| S2 | 32.44 ^c,d^ | 0.041 ^b^ | 0.96 ^b^ | 2.43 ^a^ | 0.46 ^a,b^ | 45.32 ^a,b^ |
| W2 | 57.40 ^a^ | 0.044 ^b^ | 1.13 ^b^ | 2.45 ^a^ | 0.62 ^a^ | 70.38 ^a^ |
| S1W2 | 38.00 ^b,c^ | 0.035 ^b^ | 0.70 ^b^ | 2.08 ^b^ | 0.46 ^a,b^ | 65.93 ^a,b^ |
| S2W2 | 29.65 ^d^ | 0.047 ^b^ | 0.76 ^b^ | 1.67 ^c^ | 0.41 ^b^ | 42.55 ^b^ |
| *Sig*. | *** | *** | *** | *** | * | ** |
| Species*Treatments | *** | ns | ns | ns | ns | * |

**Table S12*.*** Physiological parameters measured 90 days after the start of the experiment for *Ligustrum sinense*, *L. japonicum* ‘Texanum’, and *L. vulgare*, included SPAD units, stomatal conductance (*gₛ*, mol m⁻² s⁻¹), transpiration rate (*E*, mmol m⁻² s⁻¹), leaf vapor pressure deficit (VPD Leaf, kPa), photosystem II efficiency (PhiPS2), and electron transport rate (ETR, µmol electrons m⁻² s⁻¹). The treatments applied were as follows: control (C), salt stress at 150 mM NaCl (S1), salt stress at 300 mM NaCl (S2), water stress at 30% pot capacity (W1), water stress at 60% pot capacity (W2), combination of 150 mM NaCl and 30% pot capacity (S1W1), combination of 150 mM NaCl and 60% pot capacity (S1W2), combination of 300 mM NaCl and 30% pot capacity (S2W1), and combination of 300 mM NaCl and 60% pot capacity (S2W2). Different letters indicate significant differences among species and treatments (Tukey’s test, *p* < 0.05). ns = not significant; **p* < 0.05; ***p* < 0.01; ****p* < 0.001 denote significance levels of main effects according to ANOVA. Values within the same column followed by the same letter are not significantly different.

| **Species** | **SPAD** | **g_s_**  **mol m⁻² s⁻¹** | **E**  **mmol m⁻² s⁻¹** | **VPDLeaf**  **kPa** | **PhiPS2** | **ETR**  **µmol e⁻ m⁻² s⁻¹** |
| --- | --- | --- | --- | --- | --- | --- |
| *Ligustrum sinense* | 59.74 ^a^ | 0.020 ^b^ | 0.47 ^b^ | 2.04 | 0.52 ^a^ | 78.49 |
| *Ligustrum japonicum* ‘Texanum’ | 55.46 ^a^ | 0.085 ^a^ | 1.77 ^a^ | 1.93 | 0.37 ^b^ | 66.70 |
| *Ligustrum vulgare* | 42.69 ^b^ | 0.115 ^a^ | 2.20 ^a^ | 1.87 | 0.48 ^a,b^ | 65.90 |
| Sig. | ******* | ***** | **** | ns | *** | ns |
| Treatments |  |  |  |  |  |  |
| C | 62.12 ^a^ | 0.085 | 1.58 | 1.96 | 0.49 | 78.51 |
| S1 | 44.07 ^b^ | 0.106 | 1.92 | 1.85 | 0.40 | 56.20 |
| S2 | 36.74 ^b^ | 0.088 | 2.01 | 2.02 | 0.43 | 61.70 |
| W2 | 60.61 ^a^ | 0.111 | 2.27 | 1.95 | 0.52 | 77.77 |
| S1W2 | 39.46 ^b^ | 0.101 | 2.08 | 1.91 | 0.40 | 67.66 |
| S2W2 | 22.56 ^c^ | 0.027 | 0.50 | 1.76 | 0.39 | 50.16 |
| Sig. | *** | ns | ns | ns | ns | ns |
| Species*Treatments | *** | *** | ***** | ***** | ns | ns |
